# A detailed comparison of ΔSCF methods with the constraint-based orbital-optimized excited state method

**DOI:** 10.1038/s42004-026-02003-9

**Published:** 2026-04-22

**Authors:** Yannick Lemke, Jörg Kussmann, Christian Ochsenfeld

**Affiliations:** 1 Chair of Theoretical Chemistry, Department of Chemistry, Ludwig-Maximilians-Universität München, Munich, Germany; 2 Max-Planck-Institute for Solid State Research, Stuttgart, Germany

**Keywords:** Quantum chemistry, Method development

## Abstract

Orbital-optimized methods to variationally determine electronically excited states are becoming increasingly popular for overcoming some of the well-known shortcomings of linear-response theories. In this work we compare established ΔSCF methods with the recently proposed constraint-based orbital-optimized excited states method (COOX). In order to be able to accurately analyze the differences between both approaches, we apply the COOX method to specific orbital rotations, as defined in ΔSCF, to propose a ΔCOOX method. The main differences between these methods, as well as their performance regarding accuracy and stability, are discussed in detail. We present results for a variety of molecular systems including valence-, core-, Rydberg-, double-, and charge-transfer excitations obtained with both methods. The analysis provided in this work clearly shows that the COOX approach is superior to established ΔSCF methods in many instances, in particular regarding the overall stability for variational excited state calculations, while providing results of comparable quality.

## Abstract (web summary)

The fast and accurate computation of electronic excited states is one of the key challenges of modern quantum chemistry. In this work, the authors compare established ΔSCF methods to variationally determine electronically excited states with the recently proposed constraint-based orbital-optimized excited states method (COOX), presenting results for a variety of molecular systems including valence-, core-, Rydberg-, double-, and charge-transfer excitations, and showing that the COOX approach is superior regarding the overall stability for variational excited state calculations while providing results of comparable quality.

## Introduction

Theoretical methods to determine electronically excited states are nowadays a crucial tool to not only validate experimental data, but also to gain a deeper understanding of photo-induced chemical processes or the design of new photo-active materials. Thus, the development and improvement of such methods is an important and vibrant field of research producing multiple methods on all levels of theory from Hartree–Fock^1^ to coupled-cluster methods^2,3^, or multi-reference/active-space methods^4–10^ to handle strong correlation effects. In the last decades, linear-response time-dependent density functional theory (LR-TDDFT)^11^ emerged as the most widely used method due to its often good accuracy in combination with its comparatively low cost. However, as discussed by many authors^12–21^, there are well-known limitations to LR-TDDFT regarding, for example, the description of core-^18,19^ and charge-transfer-excitations (CT)^14–17^ or the limitation to single excitations (with respect to the ground-state)^12,13^.

To address some of these shortcomings, many research groups showed an increased interest in so-called orbital-optimized excited state methods based on DFT (OO-TDDFT, often simply referred to as “OO-DFT” in the literature. Note that we use the prefix “TD” in this instance to denote the computation of electronically excited states – which we think is missing in the “OO-DFT” nomenclature – and to provide a direct juxtaposition of orbital-optimized ("OO”) methods with linear-response ("LR”) methods, rather than to denote an explicit time-dependence, which is not present in either OO or LR methods. While many authors use the abbreviations “TDDFT” and “LR-TDDFT” interchangeably, we think this is an important distinction, as even LR-TDDFT does not involve the explicit computation of any time-dependent quantities, since it describes stationary states within the confinements of linear response). In OO-TDDFT methods, the electronically excited state is explicitly and variationally determined through the self-consistent field (SCF) procedure, most prominently in the form of ΔSCF^22^ or restricted open-shell Kohn–Sham theory (ROKS)^23–25^ with a number of available algorithmic approaches to locate stationary states, including the (initial) maximum overlap method (MOM/iMOM)^26,27^, state-targeted energy projection (STEP)^28^, and squared gradient minimization (SGM)^29^. As has been shown in many works, these methods strongly improve the description of CT- and core-excitations and even allow the description of double-excitations^29–33^ at a cost comparable to an SCF ground-state calculation, while providing a similar performance for standard small-radius Frenkel valence excitations. Furthermore, nuclear gradients can be computed via the regular ground-state tool kit without the solution of costly *Z*-vector equations. However, the applicability of these ΔSCF-like methods is also limited. Firstly, the excitation is given by clearly-defined orbital rotations, e.g., the excitation of a single electron from the highest occupied to the lowest unoccupied molecular orbital (HOMO → LUMO), preventing its use to describe excitations characterized by a mixture of multiple occupied or virtual orbitals. Of course, it is conceptually possible for ΔSCF to converge on a mixed excited state given a suitable initial guess; however, typical implementations require the user to supply the aforementioned single-orbital transitions to formulate said initial guess, rendering these states virtually inaccessible. Recent works discuss the use of fractional orbital rotations within ΔSCF^34,35^, however, mostly to overcome difficulties with degenerate orbitals and not for the description of arbitrary excitation patterns. Furthermore, these methods often suffer from slow convergence (i.e., significantly more SCF steps are required to reach convergence) or even fail to converge at all. Finally, the ΔSCF calculation may suffer from variational collapse, i.e., the calculation falls back on the initial ground-state. It should be noted that in the last decade several developments improved the stability of ΔSCF calculations, e.g., SGM^29^, direct optimization (DO-MOM)^36^, or the generalized mode following ansatz (DO-GMF)^37^, although these methods in turn require a higher computational effort and may not necessarily converge on the desired state. Several other more general ansätze such as constricted variational DFT with orbital relaxation (RSCF-CV(*n*)-DFT)^38,39^, orthogonality-constrained DFT (o-cDFT)^40^, or the closely related “variable metric” ansatz of Pham and Khaliullin^41,42^ have also shown promising results; however, so far, none of the above ansätze and algorithms could surpass the simple MOM-type methods in terms of popularity or widespread availability in quantum chemistry software – despite exhibiting superior accuracy and/or convergence behavior. We think this lack of adoption mostly originates from the complexity associated with these methods: Whereas MOM and iMOM only require a simple modification of the SCF following the diagonalization of the Fock matrix, algorithms like SGM require a complete overhaul of the central SCF structure and are thus much more cumbersome to implement into existing codes.

As an alternative ansatz for OO-TDDFT, we recently proposed the constraint-based orbital-optimized excited state method (COOX)^43–46^, which, like o-cDFT, is rooted in the formalism of constrained DFT and improves on some of the shortcomings of ΔSCF-like methods while providing the same beneficial properties. Apart from providing accurate results and a stable convergence behavior comparable to the ground-state optimization, COOX also allows to describe mixed orbital rotations, therefore enabling the calculation of arbitrary electronic excitations by forming the constraint from (simplified) TDDFT^11,47,48^ amplitudes. However, it is also possible to use specific orbital rotations as constraints (ΔCOOX), which allows us to directly compare ΔCOOX and ΔSCF-like calculations. The implementation of COOX—and cDFT in general—into existing codes is similarly straightforward as for MOM and iMOM and only requires a single modification of the existing SCF procedure between the construction and diagonalization of the Fock matrix.

In this work, we will compare ΔSCF-like methods and COOX on a theoretical level and through illustrative calculations. Therefore, we first briefly summarize the basics of the methods under investigation and analyze the key differences between them. Finally, we compare the performance of ΔCOOX and ΔSCF-like methods for different systems covering valence-, core-, and charge-transfer excitations, showing that ΔCOOX offers a substantially more stable convergence behavior at mostly comparable accuracies. Cases in which the ΔCOOX results deviate from the fully variational ΔSCF results are analyzed in detail.

## Results and discussion

### Theory

We first give a brief recap of the theory of ΔSCF-like methods, in particular the (initial) MOM method, which will be used for comparative calculations, and the theory of the ΔCOOX method. The commonalities and differences of both methods will be discussed as well as the (dis)advantages that arise from the different approaches.

In general, we aim to variationally optimize an electronically excited state that results from a well-defined orbital rotation, e.g., the excitation of a single electron from the HOMO to the LUMO. The most intuitive approach is to manually violate the Aufbau principle within each SCF step, i.e., setting the occupation numbers of *α*-HOMO to zero and *α*-LUMO to one. This is exactly what is done in the most basic ΔSCF calculations, as illustrated in Supplementary Fig. 1. A major issue with this basic approach is the required manipulation of orbital occupations within each step. During the variational optimization the order of the orbitals may change, e.g., the energy of LUMO+1 can be lower than LUMO, resulting in an incorrect orbital occupation pattern in the next SCF step. To prevent this, the (initial) MOM ansatz has been proposed^26,27^, where the overlap between the current molecular orbitals and the previous (MOM) or initial (iMOM) set of MOs,

1 $${{{\bf{O}}}}={{{{\bf{C}}}}}_{{{\rm{ref}}}}^{{{\dagger}} }{{{{\bf{SC}}}}}_{{{\rm{curr}}}},$$

is used to identify the occupied orbitals for the next iteration as those orbitals ${\phi }_{p}^{{\rm{curr}}}$ which maximize ${\sum }_{i=1}^{N}{O}_{ip}^{2}$. While MOM significantly improves the stability of ΔSCF calculations, it occasionally converges to incorrect solutions, especially for systems with orbital degeneracies. The iMOM ansatz, in contrast, ensures that a state close to the initial orbital rotation is found^27^. Although MOM/iMOM represent a significant improvement for ΔSCF calculations, they are still prone to convergence failure and sometimes even a variational collapse to the ground-state. To prevent this, the squared gradient minimization method has been developed^29^, which ensures an overall more stable optimization process, but requires additional Fock-builds and in general shows a slower convergence behavior than MOM. It should be noted that the development of more stable ΔSCF methods is still an active field of research^37^.

As mentioned, an alternative ansatz for OO-TDDFT is our COOX method based on constrained DFT (cDFT) calculations. Following the work by Van Voorhis and co-workers^49,50^, the stationary point of the energy functional, extended by an additional constraint potential term, is found as a minimum with respect to the density *ρ* and a maximum with respect to the Lagrangian multiplier *λ*_*c*_:

2 $$E[\rho ;{\lambda }_{c},{N}_{c}]={{{\rm{min}}} }_{\rho }{{{\rm{min}}}}_{{\lambda }_{c}} {E}^{0}[\rho ]+{\lambda }_{c}({{{\rm{Tr}}}}[{{{{\bf{W}}}}}_{c}{{{\bf{P}}}}]-{N}_{c})$$

Within the COOX method^43^, the constraint potential **W**_*c*_ is chosen as

3 $${{{{\bf{W}}}}}_{c}={{{\bf{S}}}}(\Delta {{{{\bf{P}}}}}^{{{\rm{virt}}}}-\Delta {{{{\bf{P}}}}}^{{{\rm{occ}}}}){{{\bf{S}}}},\,{N}_{c}=0,$$

and comprises the static (unrelaxed) parts Δ**P**^virt^ and Δ**P**^occ^ of the difference density, which can be directly constructed from the MO transition amplitudes obtained with (simplified) LR-TDDFT. As discussed in our previous works^43–46^, this ansatz allows the variational optimization of arbitrary excited states while providing a stable and fast convergence behavior. To directly compare with ΔSCF, a comparable ΔCOOX method employs the constraints obtained from the one-electron density matrices constructed from the specific MOs involved in the excitation. Following the details already outlined for our NOCI-COOX method^46^, the difference densities for the excitation ${\phi }_{i}^{\alpha }\to {\phi }_{a}^{\alpha }$ are given as the outer product of the respective MO coefficient vector with itself (note that Einstein sum convention is not used),

4 $$\Delta {P}_{\mu \nu }^{{\rm{virt}},\alpha }=\frac{1}{2}{C}_{\mu a}^{\alpha }{C}_{\nu a}^{\alpha },$$

5 $$\Delta {P}_{\mu \nu }^{{\rm{occ}},\alpha }=\frac{1}{2}{C}_{\mu i}^{\alpha }{C}_{\nu i}^{\alpha },$$

with *N*_*c*_ = 1/2 ensuring the complete depletion of ${\phi }_{i}^{\alpha }$ and population of ${\phi }_{a}^{\alpha }$. For triplet excitations, the constraint is separated into *α*- and *β*-components, i.e., ${{{{\bf{W}}}}}_{c}^{\alpha }={{{\bf{S}}}}\Delta {{{{\bf{P}}}}}^{{\mbox{virt}},\alpha }{{{\bf{S}}}}$ and ${{{{\bf{W}}}}}_{c}^{\beta }=-{{{\bf{S}}}}\Delta {{{{\bf{P}}}}}^{{\mbox{occ}},\beta }{{{\bf{S}}}}$.

Obviously, both approaches share the common goal of transferring an electron from orbital ${\phi }_{i}^{\alpha }$ to orbital ${\phi }_{a}^{\alpha }$ as indicated in Supplementary Fig. 1. While ΔSCF directly reflects the excitation picture, i.e., changing the occupation pattern, the ΔCOOX ansatz is less intuitive. Here, the Aufbau principle is preserved, and the excitation is achieved by directly manipulating the MOs. The constraint potential rather shifts the energy levels of the MOs involved in the excitation. Here, it is illustrative to consider the spectral representation of the density matrix of the ground and excited states. Within ΔSCF, the eigenvectors (MOs) of the ground-state are assumed to be a good guess for the excited state MOs, while the aim is to reoptimize the eigenvalues (occupations) for the excited state. For ΔCOOX, the eigenvalues are assumed to be valid for both ground and excited states, while only the eigenvectors are to be optimized. Of course, this distinction only holds approximately, as ΔSCF eigenvectors also change through the repeated diagonalization of the Fock matrix; a converged set of ΔSCF orbitals is thus generally distinct from the ground-state orbitals. Nevertheless, the comparison highlights the conceptual difference between ΔSCF and ΔCOOX, i.e., the former realizes orbital rotations mostly through direct modification of the occupation numbers, whereas the latter implicitly rotates the eigenvectors through the modified Lagrangian. It is noteworthy that the different objective of these methods reminds of the two classes of diagonalization-free SCF solvers, the density matrix minimization ( →  ΔCOOX) and purification methods ( →  ΔSCF)^51^.

The violation of the Aufbau principle in ΔSCF can be considered the root of the main issue of the method, i.e., convergence issues and the potential collapse to the ground-state. Since the excited state is variationally optimized, one needs to manually manipulate the occupations on top of the implicit rotation of the eigenvectors during the SCF optimization, which can result either in the collapse to the ground-state or the converging to an undesired solution. Within ΔCOOX, in contrast, we only rotate the eigenvectors to optimize the excited state while ensuring the depletion/occupation of the specific occupied/virtual orbitals, respectively, involved in the excitation.

### Low-lying excited states of benzene

**Fig. 1:**
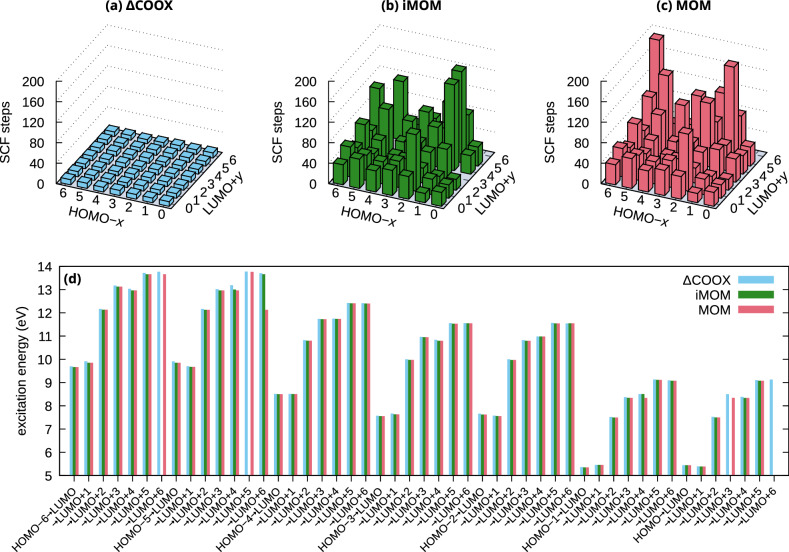
Orbital-optimized calculations of low-lying excited states of benzene. **a**–**c** Number of SCF iterations and **d** energies for all excitations HOMO − *x* → LUMO + *y* (*x*, *y* = 0, …, 6) of benzene (PBE0/def2-TZVP) using ΔCOOX, iMOM, and MOM. 4 states for iMOM and 1 state for MOM did not converge. All ΔCOOX calculations converged in 8–10 iterations.

As an illustration, we evaluated all single excitations HOMO − *x* → LUMO + *y* (*x*, *y* = 0, …, 6) of benzene using MOM, iMOM, and ΔCOOX (PBE0^52,53^/def2-TZVP^54^). The bar plot in Fig. 1 shows the excitation energies and the number of SCF steps to achieve convergence (RMS[**F**, **P**] < 10^−7^). From Fig. 1(a), it is obvious that ΔCOOX allows for a far more stable convergence within at most 10 steps, while MOM and iMOM both require many more iterations and sometimes completely fail to converge within 200 iterations (1 state for MOM, 4 states for iMOM). The excitation energies shown in Fig. 1(d) indicate that all methods converge to very similar energies for most states, while a few more strongly diverge. For example, the excitation HOMO − 5 → LUMO + 6 obtained with MOM produces the excitation HOMO − 5 → LUMO + 2, while ΔCOOX and iMOM converge to the correct result. Here, it should be noted that the spin-contaminated results are shown, i.e., no spin-purification^22,55^ has been applied. The deviation of the $\langle \hat{S}^{2}\rangle$ value from 1 was similar for all methods with a maximum deviation 0.033 for MOM/iMOM and 0.029 for ΔCOOX.

**Fig. 2:**
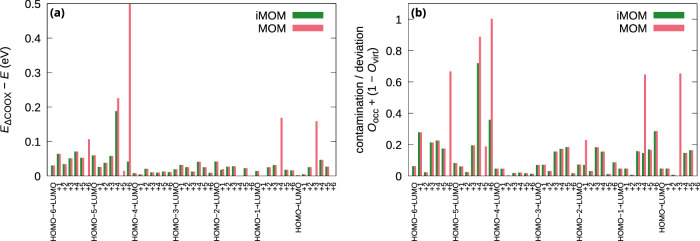
Deviations between ΔCOOX and MOM/iMOM for low-lying excited states of benzene. **a** Difference energy relative to ΔCOOX for all excitations HOMO − *x* → LUMO + *y* (*x*, *y* = 0, …, 6) of benzene (PBE0/def2-TZVP). **b** Contamination/deviation of the excited state densities (see text for details) as the sum of the projection onto the single electron density formed from the depleted occupied orbital ${\phi }_{i}^{\alpha }$ and the deviation from one of the projection onto the corresponding density formed from the filled virtual orbital ${\phi }_{a}^{\alpha }$. By definition, contamination/deviation for ΔCOOX is zero. Unconverged MOM/iMOM results are neglected.

Regarding the deviation of MOM/iMOM compared to ΔCOOX, we also show the energy difference in Fig. 2(a), where it is noteworthy that ΔCOOX always predicts larger excitation energies. To analyze the potential source of these deviations, we determined the purity of the excitation by evaluating the overlap of the excited state densities **P**^exc^ with the single-electron densities formed from the orbitals involved in the excitations, which are also used for the construction of the corresponding constraint potential,

6 $${O}_{{\rm{occ}}}=2Tr[{{{{\bf{P}}}}}^{{{\rm{exc}}}}{{{\bf{S}}}}\Delta {{{{\bf{P}}}}}^{{\rm{occ}},\alpha }{{{\bf{S}}}}]$$

7 $${O}_{{\rm{virt}}}=2Tr[{{{{\bf{P}}}}}^{{{\rm{exc}}}}{{{\bf{S}}}}\Delta {{{{\bf{P}}}}}^{{\rm{virt}},\alpha }{{{\bf{S}}}}],$$

where a factor of 2 is introduced for normalization since Δ**P** includes an implicit factor of 1/2 (recall equations (4) and (5)). The contamination/deviation plotted in Fig. 2(b) is the sum of *O*_occ_ and 1 − *O*_virt_; the individual values of *O*_occ_ and *O*_virt_ can be found in Supplementary Fig. 2. By construction, ΔCOOX always yields zero contamination/deviation as the constraint completely removes any trace of ${\phi }_{i}^{\alpha }$ and occupies ${\phi }_{a}^{\alpha }$ with one electron. Ignoring the outlier of the HOMO − 5 → LUMO + 6 excitation, where MOM produces the wrong state, we see that the patterns of both plots in Fig. 2 closely match, i.e., the larger the contamination/deviation, the larger the difference in excitation energies as compared to ΔCOOX. This may be understood as either the presence of mixed excitation patterns or a result of relaxation of the populated virtual orbital. Note that the terms “contamination” and “deviation” are not to be mistaken for indicators of qualitative or quantitative correctness, i.e., a small amount of contamination and deviation does not imply an incorrect excited state. Instead, these measures merely quantify how much a given unconstrained stationary point deviates from the specified initial guess. As stated before, small values of contamination/deviation can arise as a consequence of (desired) orbital relaxation and generally lead to more accurate excitation energies, whereas values close to unity indicate that the ΔSCF calculation converged towards an undesired state. However, this raises an important question for stationary ΔSCF solutions: What are the bounds on contamination/deviation for which one can be confident that the intended state has been obtained (i.e., how closely does the excited state need to match the specified excitation pattern)? Is an intermediate amount of contamination/deviation, say 0.5, to be interpreted as a consequence of orbital relaxation, or is there a different stationary point that more closely resembles the requested excited state, which the SCF failed to converge towards? While ΔCOOX always satisfies the requested excitation by definition, we do not think that there is a systematic way to decide these questions in the case of ΔSCF. Therefore, ΔCOOX should, at the very least, be considered as a starting point to search for variational solutions in the vicinity.

The results obtained for benzene indicate that ΔCOOX offers a far more stable and efficient way to variationally determine explicit electronically excited states. Due to the adherence to the Aufbau principle, it is impossible to suffer from a variational collapse back to the ground-state, while the explicit constraint potential ensures that the requested state is obtained by construction, whereas ΔSCF methods may occasionally converge towards a different state. It should further be stressed that the superior performance of ΔCOOX is also independent of the chosen functional. The analogous results for PBE^56^/def2-TZVP and HF/def2-TZVP can be found in Supplementary Figs. 3–5 and 6–8, respectively; a comprehensive summary of all calculations is tabulated in Supplementary Data 2.

In the next sections, we present further illustrative calculations covering valence-, core-, and charge-transfer excitations as well as systems in complex environments. The results of MOM, iMOM, and ΔCOOX will be compared with respect to both accuracy and stability. While we also investigated the projector-based MOM and iMOM variants of Hratchian and co-workers (PMOM and PiMOM, respectively)^57^, we found that they only sporadically improved convergence in some cases while worsening convergence in other cases; therefore, we will not be discussing PMOM and PiMOM for the remainder of this work. Unless stated otherwise, the following results are spin-purified using the approximate projection formula^55^

8 $${E}_{{\rm{S}}}=\frac{{\langle \hat{S}^{2}\rangle }_{{{\rm{T}}}}{E}_{{{\rm{BS}}}}-{\langle \hat{S}^{2}\rangle }_{{{\rm{BS}}}}{E}_{{{\rm{T}}}}}{{\langle \hat{S}^{2}\rangle }_{{{\rm{T}}}}-{\langle \hat{S}^{2}\rangle }_{{{\rm{BS}}}}}$$

with the subscripts S, T, and BS denoting the singlet, triplet, and broken-symmetry states, respectively. Typically, we have ${\langle \hat{S}^{2}\rangle }_{{{\rm{T}}}}\approx 2$ and ${\langle \hat{S}^{2}\rangle }_{{{\rm{BS}}}}\approx 1$, thus, equation (8) simplifies to

9 $${E}_{{{\rm{S}}}}\approx 2{E}_{{{\rm{BS}}}}-{E}_{{{\rm{T}}}}.$$

To ensure the validity of equation (9) as much as possible, we perform all triplet calculations within the constrained unrestricted Hartree–Fock (CUHF) framework^58^.

### Valence and Rydberg states of beryllium

In Table 1, we show exemplary excitation energies for the valence 2s2p and Rydberg 2s3s excitations in the beryllium atom computed with ΔCOOX and iMOM using the def2-TZVPPD basis set^54,59^. We include data computed from four PBE-based functionals with varying amounts of exact exchange (EXX), namely PBE itself (no EXX)^56^, PBE0 (25% EXX)^52,53^, PBE50 (50% EXX)^60^, and LRC-*ω*PBE^61^ (0% short-range EXX, 100% long-range EXX, $\omega =0.3\,{a}_{0}^{-1}$). Overall, ΔCOOX and iMOM provide energies of similar quality for the four investigated excitations, though a number of differences stand out: For iMOM, the triplet excitation energies seem mostly insensitive to the amount of exact exchange included in the density functional approximation with the only notable exception being the 2s3s state computed with LRC-*ω*PBE, whereas the singlet energies generally increase with larger EXX admixture. While iMOM matches the experimental values for the 2s3s Rydberg state more closely, it is noteworthy that the contamination/deviation shown in parentheses increases substantially as more EXX is included. This indicates that describing the excited state through a single orbital transition may not be appropriate; in particular, the deviation 1 − *O*_virt_ increases while the contamination *O*_occ_ remains close to zero. Therefore, a straightforward modification suggests itself for ΔCOOX wherein we employ the same core–valence separation (CVS) scheme as used for core-excitations^44^, i.e., we perform a CVS-TDA-TDDFT calculation in which the occupied space is restricted to the orbital which should be depleted (in this case the HOMO) and use the resulting transition amplitudes **X** to construct the difference density **ΔP** that is then used for the constraint potential in the subsequent COOX calculation. Using this approach with the LRC-*ω*PBE functional, we obtain respective triplet and singlet excitation energies of 2.28 eV and 5.40 eV for the 2s2p state and 6.44 eV and 6.98 eV for the 2s3s state. It should also be noted that a higher deviation in the ΔCOOX scheme can be permitted by using a looser tolerance value for $| Tr[{{{{\bf{W}}}}}_{c}{{{\bf{P}}}}]-{N}_{c}|$ in the determination of *λ*_*c*_. For example, applying a relatively loose threshold of 10^−2^ as opposed to the usual 10^−10^ for ΔCOOX/LRC-*ω*PBE yields 2.78 eV/4.76 eV for the 2s2p states and 6.51 eV/6.81 eV for the 2s3s states, respectively. Again, the ΔCOOX approach exhibits superior convergence behavior, converging all but one state in 5–8 iterations, whereas iMOM behaves far more erratically, requiring only 5 iterations in some cases and up to 80 iterations in others.

**Table 1:** Triplet (T) and singlet (S) excitation energies for the 2s2p and 2s3s configurations of beryllium

state	exp.		functional	ΔCOOX		iMOM			
	T	S		T	S	T		S	
2s2p (valence)	2.73	5.28	PBE	2.37	4.10	2.31	(9.4 × 10^−3^)	4.15	(1.6 × 10^−3^)
			PBE0	2.51	4.31	2.30	(4.1 × 10^−2^)	4.41	(1.6 × 10^−2^)
			PBE50	2.79	4.55	2.27	(1.0 × 10^−1^)	4.67	(5.1 × 10^−2^)
			LRC-*ω*PBE	3.18	4.72	2.28	(1.7 × 10^−1^)	4.65	(1.2 × 10^−1^)
2s3s (Rydberg)	6.46	6.78	PBE	6.28	6.39	6.23	(6.5 × 10^−3^)	6.43	(5.7 × 10^−3^)
			PBE0	6.34	6.52	6.25	(1.0 × 10^−2^)	6.55	(5.0 × 10^−3^)
			PBE50	6.40	6.65	6.28	(1.5 × 10^−2^)	6.67	(5.7 × 10^−3^)
			LRC-*ω*PBE	6.63	6.84	6.43	(2.4 × 10^−2^)	6.80	(1.4 × 10^−2^)

All energies in eV. Calculations were performed using the def2-TZVPPD basis set. Values in parentheses indicate the iMOM contamination/deviation *O*_occ_ + (1 − *O*_virt_) from an ideal constraint value.

### Double-excitations

A further advantage of orbital-optimized excited state methods compared to LR-TDDFT is the possibility to determine double-excitations. To illustrate the accuracy and performance of our COOX ansatz, we evaluate a subset of double-excitations taken from the benchmark set published by Loos et al.^62,63^. In Table 2 the results for ΔCOOX and iMOM using the def2-TZVP basis and different functionals are shown, also including the *ω*B97X functional^64^ with 15.771% short-range EXX and 100% long-range EXX. The geometries are taken from Hait and Head-Gordon^29^.

**Table 2:** Double-excitation energies for selected systems

	iMOM				ΔCOOX				
	PBE	PBE0	LRC-*ω*PBE	*ω*B97X	PBE	PBE0	LRC-*ω*PBE	*ω*B97X	TBE^b^
Be	7.046	7.287	7.494	7.431	7.076	7.458	8.015	8.166	7.151
HNO	4.182	4.284	4.219	4.287	4.351	4.365	4.335	4.397	4.333
HCHO	9.887^a^	10.198	10.145	10.308	10.951	10.839	10.915	10.958	10.426
C_2_H_4_	11.834	12.359	12.403	12.613	11.866	12.458	12.642	12.903	12.899
CH_3_NO	4.652	4.718	4.663	4.713	4.842	4.811	4.799	4.852	4.732
Glyoxal	4.981	5.898	6.085	6.544	5.061	5.963	6.221	6.687	5.492
Pyrazine	7.544	8.488	8.684	9.101	7.634	8.567	8.814	9.253	7.904

All energies in eV. Energies for iMOM and ΔCOOX were computed using the def2-TZVP basis set.

^a^MOM energy: 50.744 eV.

^b^Theoretical best estimates best estimates (TBE) of Kossoski et al.^63^ are also shown.

Apart from the MOM-PBE result for formaldehyde, which converged to an undesired state, all methods produce fairly similar results, while deviations between MOM/iMOM and ΔCOOX correlate with contamination/deviation as has been discussed before. Again, it should be stressed that the convergence behavior for ΔCOOX is consistently more stable than iMOM/MOM with at most 9 iterations to achieve convergence. The detailed results including the number of necessary SCF steps and values for contamination/deviation can be found in Supplementary Data 3.

### Charge-transfer excitations

The description of charge-transfer (CT) states, especially long-range CT, represents another widely known challenge for LR-TDDFT^14–16^, and the superior performance of orbital-optimized methods for such states is well-documented in the literature^33,65^. In a previous work^43^, we observed that COOX in the restricted formalism, much like LR-TDDFT approaches, requires the inclusion of long-range EXX to avoid contamination arising from energetically lower-lying local excitations. Here, we reassess the performance using a modified version of the ΔCOOX ansatz: If we were to use the *α*-only constraint as defined in equations (4) and (5), the SCF may converge to an energetically lower state of two triplets with opposite magnetization that could be described as ^1^TT rather than the desired (broken-symmetry) ^1^DD state, owing to the backflow of a *β*-electron from the acceptor to the donor. We further illustrate and discuss this issue in Supplementary Fig. 9 and Supplementary Note 1. To obtain the desired CT state, we augment the constraint through the appropriate *β*-projectors, i.e.,

10 $${{{{\bf{W}}}}}_{c}^{\alpha }={{{\bf{S}}}}(\Delta {{{{\bf{P}}}}}_{{{\rm{A}}}}^{{\rm{virt}},\alpha }-\Delta {{{{\bf{P}}}}}_{{{\rm{D}}}}^{{\rm{occ}},\alpha }){{{\bf{S}}}}$$

11 $${{{{\bf{W}}}}}_{c}^{\beta }={{{\bf{S}}}}(\Delta {{{{\bf{P}}}}}_{{{\rm{A}}}}^{{\rm{occ}},\beta }-\Delta {{{{\bf{P}}}}}_{{{\rm{D}}}}^{{\rm{virt}},\beta }){{{\bf{S}}}},$$

where the subscripts D and A indicate the donor and acceptor molecule, respectively. Since this constraint involves two electrons, the corresponding value of *N*_*c*_ is 1 rather than 1/2.

**Fig. 3:**
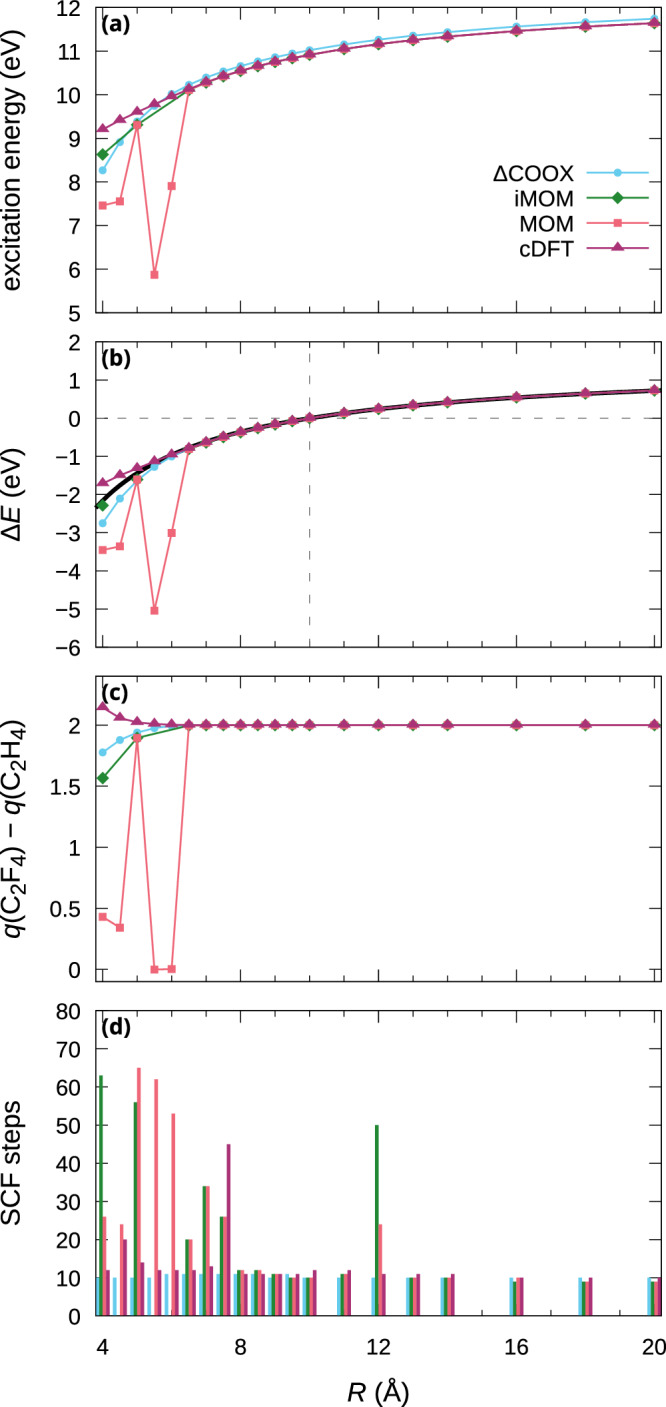
Intermolecular charge-transfer in C_2_H_4_–C_2_F_4_ at different intermolecular separations. **a** Vertical charge-transfer excitation energies, **b** relative energies aligned at *R* = 10 Å with asymptotic  −1/*R* curve in black, **c** Mulliken charge differences, and **d** number of SCF steps for C_2_H_4_–C_2_F_4_, computed using LRC-*ω*PBE/def2-TZVP.

Using equations (10) and (11), we computed excitation energies for the ethylene–tetrafluoroethylene system at different intermolecular distances. Since the singlet and triplet CT states become degenerate at large separation, we forego the spin-purification procedure and instead show the broken-symmetry energies in Fig. 3 for the LRC-*ω*PBE functional (results for other functionals are included in Supplementary Fig. 10). Besides the MOM and iMOM results, we also include data for broken-symmetry cDFT obtained using two constraints on the respective charge and spin difference of the two monomers. As can be seen, MOM and iMOM suffer from numerical instabilities at short separations; in particular, iMOM completely fails to converge for a number of cases, whereas MOM occasionally collapses to local Frenkel excitations (Fig. 3(c)). Furthermore, both approaches require a substantial number of iterations to achieve convergence at small *R* (Fig. 3(d)). It can also be observed that cDFT yields a larger amount of charge separation and thus higher excitation energies at small distances, which highlights the questionable validity of spatial constraint potentials for fragments which are not well-separated. At large separations, all methods converge in up to 10 iterations and exhibit the correct asymptotic  −1/*R* behavior as shown in Fig. 3(b). In Supplementary Fig. 11, we show the difference densities computed at 6 Å and 10 Å separation to further illustrate these findings: For *R* = 6 Å, iMOM fails to converge, and MOM collapses onto a local excitation on the C_2_F_4_ monomer, whereas cDFT and ΔCOOX agree nicely both in terms of difference density and excitation energy. At *R* = 10 Å, all methods produce nearly identical difference densities and excitation energies, with only ΔCOOX deviating slightly, likely as a result of contamination/deviation as discussed in previous sections.

While ΔCOOX conforms to the expected  −1/*R* behavior for LRC-*ω*PBE, the same is not true for the PBE- and PBE0-based ΔCOOX results, where we need to employ Fermi smearing to achieve stable convergence, which reintroduces the problem of charge backflow as shown in Supplementary Fig. 10. Thus, it appears as though the inclusion of long-range EXX remains a crucial factor for accurate COOX-based charge-transfer excitations. In contrast, the ΔSCF-based methods show remarkably little functional-dependence for CT states. This phenomenon has already been noted by other authors^65^, but to the best of our knowledge its origins have not yet been studied in detail. To understand whether this lack of functional dependence arises as a result of the underlying physics of non-Aufbau solutions or—as is often the case in DFT—through a fortuitous cancellation of errors would certainly be illuminating and should be investigated in future research.

To compare with higher-level methods, we separately computed excited states of the C_2_H_4_–C_2_F_4_ system at separations of 3.5 Å and 5.0 Å using the optimized geometries of Szalay and co-workers^66^, which we list in Table 3 alongside the ADC(2) and CCSDT-3 values from the same work^66^. At the shorter separation of *R* = 3.5 Å, where there is no discernible CT state, iMOM again exhibits convergence problems, so that a direct comparison with ΔCOOX is challenging. In general, both orbital-optimized methods yield lower excitation energies than the higher-level reference methods, though this may be an artifact of the smaller basis set used in the ADC(2) and CCSDT-3 calculations^66^ (as noted by the authors, changing the basis set from cc-pVDZ to cc-pVTZ lowers the CCSDT-3 energy for the CT state from 10.56 eV to 10.19 eV). At the larger separation, the CT state for iMOM again appears considerably less sensitive to the choice of functional than is the case for ΔCOOX. Interestingly, the same applies neither for the local *π* → π* states at *R* = 5.0 Å nor for the three ^1^B_1_ states at *R* = 3.5 Å, for which iMOM exhibits similar or even greater functional dependence than ΔCOOX.

**Table 3:** Spin-purified iMOM and ΔCOOX excitation energies for the C_2_H_4_–C_2_F_4_ system at two intermolecular separations

	iMOM			ΔCOOX			Reference^a^	
State	PBE	PBE0	LRC-*ω*PBE	PBE	PBE0	LRC-*ω*PBE	ADC(2)	CCSDT-3
*R* = 3.5 Å								
1 ^1^B_1_	5.14	7.38	6.83	6.83	7.02	7.27	7.97	8.09
2 ^1^B_1_	6.68	—	7.19	6.39	7.06	7.39	8.79	8.84
3 ^1^B_1_	—	—	7.37	6.34	7.38	7.51	8.81	9.02
*R* = 5.0 Å								
C_2_H_4_* π* → π^*^	6.60	7.28	7.37	6.55	7.16	7.27	8.54	8.61
C_2_F_4_* π* → π^*^	6.47	7.57	7.47	6.40	7.46	7.39	8.71	8.99
CT	8.72	9.23	9.25	7.34	7.62	9.20	10.28	10.56

All excitation energies in eV. Energies for iMOM and ΔCOOX were computed using the def2-TZVP basis set; missing entries mean that the calculation did not converge.

^a^Reference values from Kozma et al. ^66^ computed with the cc-pVDZ basis set invoking the frozen-core approximation.

**Fig. 4:**
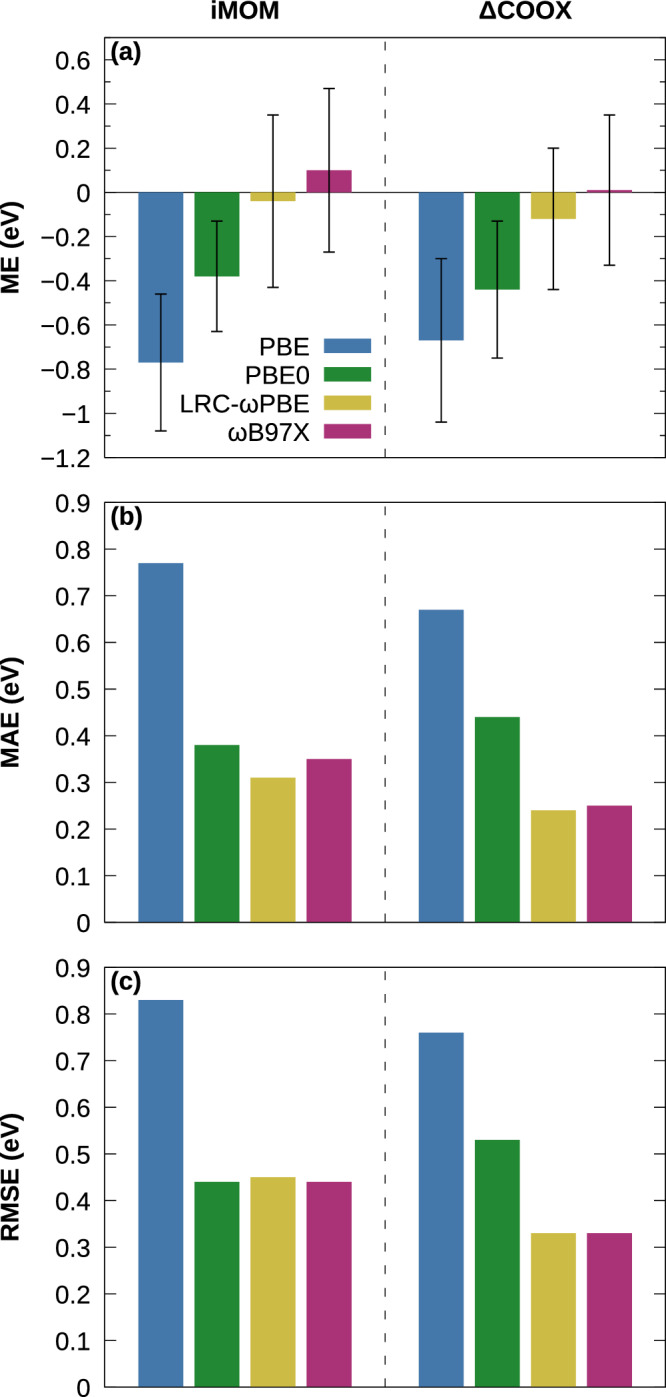
Performance for the intramolecular charge-transfer benchmark from the QUEST database. **a** Mean error (ME) with standard deviation (error bars), **b** mean absolute error (MAE), and **c** root-mean-squared error (RMSE), computed using iMOM and ΔCOOX with the PBE, PBE0, LRC-*ω*PBE, and *ω*B97X functionals and the def2-TZVP basis set. For iMOM, a number of states did not converge; these states are excluded from the statistics (see Supplementary Table 3).

In Fig. 4, we further show the performance of ΔCOOX and iMOM for a benchmark of intramolecular CT excitations taken from the QUEST database^67^ for various functionals. Note that a separation of constraints along the lines of equations (10) and (11) often appears unnecessary for intramolecular CT; for small systems such as those in the QUEST database, there is no competing superposition of local single excitations like in intermolecular CT; for larger systems—in which there may exist well-separated donor and acceptor moieties with local excitations—the potentially problematic ^1^TT-like double-excitation has proven to be appreciably high in energy compared to the intramolecular CT state for most systems we have investigated so far. There are, however, some systems with sufficiently low-lying local excitations, in which case the separation of constraints is required; an example for this is provided for the zincbacteriochlorin–bacteriochlorin complex^15^ in the Supplementary Fig. 12 and Supplementary Table 1. In practice, this is straightforward due to the spatial separation between donor and acceptor moieties, and the application of a split constraint does not appear to be detrimental in cases where the local excitations are comparatively high in energy, e.g., in medium-sized thermally activated delayed fluorescence (TADF) emitters (see Supplementary Fig. 13, Supplementary Table 1, and Supplementary Note 2). The excited state obtained through ΔCOOX can easily be verified through two straightforward metrics, i.e., the $\langle \hat{S}^{2}\rangle$ expectation value and the projection of the excited-state density onto the virtual space of the ground-state given by

12 $${n}_{{{\rm{exc}}}}=Tr[{{{{\bf{P}}}}}^{{{\rm{exc}}}}({{{\bf{S}}}}-{{{\bf{S}}}}{{{{\bf{P}}}}}^{0}{{{\bf{S}}}})],$$

which amounts to the number of electrons excited into the (original) virtual space. For the charge-transfer state, one obtains $\langle {\hat{S}}^{2}\rangle \approx 1$ and *n*_exc_ ≈ 1, whereas the ^1^TT-like double-excitation results in $\langle {\hat{S}}^{2}\rangle \approx 2$ and *n*_exc_ ≈ 2. Returning to Fig. 4, with the exception of PBE0 and the mean error for LRC-*ω*PBE, ΔCOOX generally yields slightly lower errors than iMOM, and we again observe a trend towards higher excitation energies for increasing admixture of EXX. Notably, the functional independence exhibited by iMOM for long-range intermolecular CT cannot be observed here. The best overall performance is achieved by ΔCOOX with the long-range corrected hybrid functionals, with respective mean, mean absolute, and root-mean-squared errors (ME, MAE, and RMSE) of −0.12 eV, 0.24 eV, and 0.33 eV for LRC-*ω*PBE and 0.01 eV, 0.25 eV, and 0.33 eV for *ω*B97X. The discrepancies for PBE0 and the mean error of LRC-*ω*PBE originate from the *β*-dipeptide system, which is excluded for iMOM due to convergence failure and is responsible for the largest deviations for ΔCOOX with −1.28 eV ($7 {{\rm{A}}}^{\prime}$, PBE0) and −0.90 eV (10 A*″*, LRC-*ω*PBE) compared to the theoretical best estimate, respectively. Excluding dipeptide and *β*-dipeptide from the statistical analysis, the error metrics of ΔCOOX@LRC-*ω*PBE are further reduced to an ME of −0.05 eV, MAE of 0.18 eV, and RMSE of 0.23 eV. The individual excitation energies are listed in Supplementary Tables 2 (spin-contaminated) and 3 (spin-purified); relevant metrics are defined in Supplementary Note 3.

### Core-excitations

**Fig. 5:**
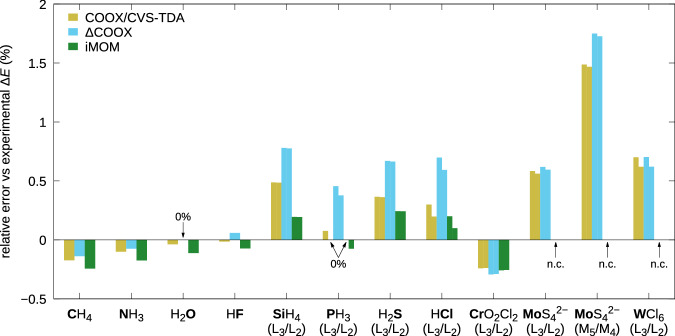
Performance for a selection of K-, L-, and M-edge excitations. Relative errors for core-excitation energies computed with the PBE0 functional. Basis sets used: aug-pcX-2^82^ for CH_4_ through HCl, aug-cc-pwCVTZ-DK^83,84^ for CrO_2_Cl_2_ and MoS_4_^2−^, ma-ZORA-def2-TZVPP/SARC-ZORA-TZVPP^85^ for WCl_6_. “0%” means the excitation energy is matched exactly up to the experimental precision available; “n.c.” means the calculation did not converge.

In a previous work^44^, we showed that COOX affords excellent results for core-excitations, improving on the already very good results obtained with iMOM in terms of excitation energies and especially algorithmic stability for heavier atoms. In Fig. 5, we revisit a selection of species from across the periodic table. Due to the vastly different excitation energies, which span three orders of magnitude, we show relative errors in percent; for absolute values see Supplementary Table 4. Calculations were performed using the scaled version of the scalar-relativistic zero-order regular approximation Hamiltonian^68^ with the effective potential of van Wüllen^69^. For L- and M-edge excitations, spin–orbit coupling is accounted for in a semiempirical fashion using a matrix representation of the spin–orbit coupling operator as discussed by Hait and Head-Gordon for L-edge excitations^30^ and in our work for M-edge excitations^44^. Spin purification is applied for all values using the appropriate triplet energies obtained with application of the CUHF formalism. For the basis sets used, see the caption of Fig. 5. Overall, all tested methods yield excitation energies to a good degree of accuracy, only exceeding relative errors of 1% for the M-edge excitation of MoS_4_^2−^. In most cases, ΔCOOX leads to slightly higher excitation energies than either iMOM or COOX using CVS-TDA difference densities, which aligns with comparatively large contamination/deviation values for both (see Supplementary Table 4). Once again, the COOX-based approaches exhibit better numerical robustness, with iMOM failing to converge for MoS_4_^2−^ and WCl_6_ and otherwise requiring more SCF iterations to achieve convergence.

### Excited states in complex environments

A further feature of the COOX ansatz is the straightforward treatment of complex molecular or bulk environments such as proteins or explicit solvation shells. Our constraint-embedding scheme, termed “e-COOX”^45^ enables a black-box computation of the state of interest while retaining the full quantum-mechanical treatment of the environment at low computational cost. In e-COOX, the constraint-defining difference densities Δ**P**^virt^ and Δ**P**^occ^ are obtained from the isolated subsystem and subsequently embedded into the full quantum system through Gram–Schmidt orthogonalization of the subsystem MOs such that

13 $$\forall \,i\in {{{\rm{occ}}}},a\in {{{\rm{virt}}}}:{\phi }_{i}^{{{\rm{sub}}}}\perp {\phi }_{a}^{{{\rm{full}}}},\,{\phi }_{a}^{{{\rm{sub}}}}\perp {\phi }_{i}^{{{\rm{full}}}},\,{\phi }_{i}^{{{\rm{sub}}}}\perp {\phi }_{a}^{{{\rm{sub}}}}.$$

**Fig. 6:**
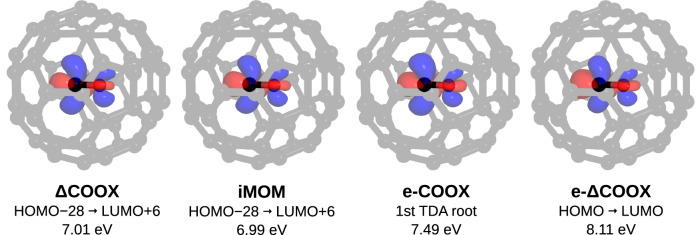
Difference densities and excitation energies for the *π* → π* excitation of carbon monoxide inside a C_60_ fullerene. For ΔCOOX, iMOM in the full system, the relevant transition is HOMO − 28 → LUMO + 6. Using the e-COOX embedding scheme, the transition is given by the first TDA root for regular e-COOX and by the HOMO → LUMO transition for e-ΔCOOX. All difference densities are drawn at an isovalue of 0.01 a.u.

We illustrate the advantage of this approach for the HOMO → LUMO excitation of carbon monoxide enclosed in a C_60_ fullerene. While this system is admittedly highly artificial, it clearly shows the difficulty of identifying the desired excitation when the environment is treated quantum-mechanically: For PBE/def2-SVP, the excitation of interest is obtained as the 455th simplified TDA root, or alternatively as the transition HOMO − 28 → LUMO + 6. As can be seen in Fig. 6, the e-COOX and e-ΔCOOX treatments afford very similar difference densities to ΔCOOX and iMOM in the full system, although the excitation energies are slightly higher. It should again be highlighted that for e-COOX and e-ΔCOOX, the states in question are easily obtained as the first TDA root and the subsystem HOMO → LUMO excitation, respectively, enabling black-box-type applications for systems in complex environments without the need for time-consuming manual orbital assignments.

### ΔCOOX as a preconditioner for MOM and iMOM

**Fig. 7:**
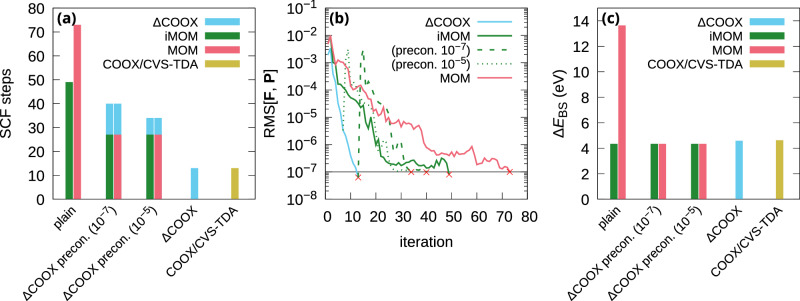
Convergence behavior and accuracy for the HOMO → LUMO excitation of nitrobenzene with and without ΔCOOX preconditioning. **a** Number of SCF steps, **b** SCF convergence, and **c** broken-symmetry excitation energies. Values in parentheses indicate the SCF convergence criterion for ΔCOOX preconditioning (see text for further details); computed at the PBE0/def2-TZVP level. Preconditioned MOM calculations in (**b**) are excluded since they coincide with preconditioned iMOM.

As a final consideration, we investigate the idea of using ΔCOOX as a preconditioner for a subsequent MOM or iMOM calculation. Since we have shown ΔCOOX excited states to often be similar to ΔSCF-type results, the optimized ΔCOOX orbitals should serve as a much better initial guess for MOM or iMOM than the manually interchanged ground-state orbitals. In Fig. 7, we show the effect of this ansatz for the HOMO → LUMO excitation of nitrobenzene computed with PBE0/def2-TZVP using the optimized vacuum geometry from Mewes et al.^70^. Whereas iMOM converges to the desired state in 49 iterations, MOM requires 73 iterations and converges to an incorrect doubly-excited state. Applying the ΔCOOX preconditioning, we can not only reduce the number of required MOM/iMOM iterations to 27, but also restore the correct excited state for MOM. This even holds when running the preceding ΔCOOX calculation with a looser convergence criterion of RMS[**F**, **P**] < 10^−5^, which reduces the number of required ΔCOOX steps from 13 to 7. Nevertheless, the pure ΔCOOX approach using either a single-orbital transition or a CVS-TDA difference density is still clearly superior in terms of required SCF steps and closely matches the iMOM excitation energies. For the COOX/CVS-TDA calculation, the virtual space was restricted to the LUMO only while allowing transitions from the complete occupied space, as the full TDA-TDDFT amplitudes are characterized predominantly by HOMO − 1 → LUMO and HOMO → LUMO. Unfortunately, a full comparison of spin-purified excitation energies to higher-level methods is not possible, as the corresponding triplet state fails to converge for iMOM and MOM even when using ΔCOOX preconditioning. On the other hand, stable convergence is achieved with COOX-based methods, and we observe overall good agreement with the ADC(3) excitation energy of 4.67 eV given in the literature^70^, with respective energies of 4.68 eV for ΔCOOX, 4.79 eV for COOX/CVS-TDA, and 4.84 eV for the restricted scaled-COOX method using full TDA-TDDFT difference densities. Another potential application of this ansatz is in combination with the e-COOX embedding scheme discussed above: Using the converged e-ΔCOOX state shown in Fig. 6, a subsequent iMOM calculation set to maximize the overlap with the ΔCOOX orbitals converges to precisely the same iMOM state as shown in Fig. 6 (6.99 eV) in 15 rather than 20 steps without the need to manually identify the transition.

## Conclusions

We presented a detailed comparison between established ΔSCF methods with our recently proposed COOX method, in particular, the ΔCOOX variant that aims for specific orbital rotations directly comparable to ΔSCF. From the analysis presented in this work, it is apparent that the explicit violation of the Aufbau principle in ΔSCF results in an unstable optimization, i.e., an often strongly inflated number of necessary steps, convergence failure, obtaining undesired results, or a collapse back onto the ground-state.

In conclusion, it is apparent that our ΔCOOX method is highly competitive and in many cases clearly superior to established ΔSCF methods. Based on our findings, we would argue that ΔCOOX should be considered the method of choice for the evaluation of a vast majority of variationally optimized electronically excited states defined through single-orbital transitions and methods building thereupon, such as non-orthogonal configuration interaction^46^. Unfortunately, we cannot directly compare ΔCOOX to some of the more sophisticated algorithms for ΔSCF, which, likewise, have been shown to outperform MOM and iMOM, due to a lack of availability and an implementation overhead that would exceed the scope of this work. However, based on literature results for some of the systems also studied in this work, we would anticipate ΔCOOX to offer faster convergence through both fewer required SCF iterations and a reduced cost per SCF iteration, with similar implications for the accuracy as we have observed compared to MOM and iMOM. Moreover, an adaptation of the restricted open-shell Kohn–Sham formalism^23,24^ for ΔCOOX should be straightforward and could be an interesting future avenue. However, we would like to emphasize that our original COOX method^43^ using constraints from (simplified) TDDFT transition coefficients still represents a more flexible, effective, and reliable way to determine orbital-optimized excited states. While it features the same advantages as ΔCOOX, it allows to describe arbitrary electronic excitations, including those involving multiple occupied/virtual orbitals, and has been shown to rectify some of the deficiencies of the underlying LR-TDDFT constraint potential, e.g., in the description of partial double-excitations or core-excitations^43^. For more complex scenarios, such as states with a highly mixed excitation character, the non-orthogonal configuration interaction ansatz offers a good compromise between accuracy and computational efficiency. Finally, we would like to reiterate that the implementation of COOX (and by extension ΔCOOX) is straightforward, especially if cDFT routines are already implemented, and is compatible with common convergence acceleration techniques such as the widely used direct inversion in the iterative subspace (DIIS)^71^ without requiring further modifications of the SCF procedure.

## Methods

All calculations were performed using our in-house FermiONs++ program package^72–74^. Exchange–correlation contributions for Kohn–Sham DFT were computed with the LIBXC library^75,76^ using the modified Becke grids of Laqua et al.^77^ (in particular, gm5). For exact exchange contributions, the sn-LinK method was used^78,79^. Calculations were performed using tight integral screening thresholds (10^−10^ a.u.) and, unless specified otherwise, an SCF convergence threshold of RMS[**F**, **P**] < 10^−7^. For COOX and ΔCOOX, the Lagrange multiplier *λ*_*c*_ was determined through the TOMS748 bracketing algorithm^80^ with a threshold of $| Tr[{{{{\bf{W}}}}}_{c}{{{\bf{P}}}}]-{N}_{c}| < 1{0}^{-10}$. Pulay’s direct inversion in the iterative subspace (DIIS)^71^ convergence acceleration was used in all calculations, where the two-step DIIS procedure outlined by Kaduk et al.^50^ was used for cDFT-based methods. Spatial potentials for regular cDFT calculations were computed through Becke’s integration scheme^81^. If not stated otherwise, broken-symmetry singlet excitation energies were purified using the approximate projection formula of Yamaguchi et al.^55^ For triplet states, the CUHF formalism^58^ was applied to minimize spin contamination.

A variety of single-reference methods were sampled, i.e., Hartree–Fock, PBE^56^, PBE0^52,53^, PBE50^60^, LRC-*ω*PBE^61^, and *ω*B97X^64^. Most calculations used the “Karlsruhe def2” basis sets^54,59^ with the exception of core-excitations, where we employed the aug-pcX-2^82^, aug-cc-pwCVTZ-DK^83,84^, or ma-ZORA-def2-TZVPP/SARC-ZORA-TZVPP^85^ basis sets depending on availability for the atoms in question (i.e., aug-pcX-2 if available for all atoms, otherwise aug-cc-pwCVTZ-DK if available for all atoms, otherwise ma-ZORA-def2-TZVPP/SARC-ZORA-TZVPP). For core-excitations we further used the scalar-relativistic scaled-ZORA Hamiltonian^68^ with the effective potential developed by van Wüllen^69^ alongside the semiempirical spin–orbit coupling treatment for L-edge^30^ and M-edge excitations^44^.

## Supplementary information

Supplementary Information

Description of Additional Supplementary Files

SI Data1

SI Data2

SI Data3

## Data Availability

All data supporting our findings is provided in the main manuscript and the Supplementary Information. Cartesian coordinates for all studied systems can be found in Supplementary Data 1. Supplementary Data 2 and 3 contain detailed listings of excitation energies, number of SCF iterations, contamination/deviation values, etc., for all calculations performed on benzene and all studied double-excitations, respectively. Numerical data, as well as Gnuplot and PyMol source code to reproduce the graphics presented in this work, have been made available on Zenodo^86^.

## Code availability

COOX and ΔCOOX are implemented in our in-house FermiONs++ program package which is currently being prepared for release.
