# Supplementary material for: A detailed comparison of ΔSCF methods with the constraint-based orbital-optimized excited state method
Source: Commun Chem. 2026 Apr 22;9:162. doi: 10.1038/s42004-026-02003-9 (PMC13102931; doi:10.1038/s42004-026-02003-9)
Supplement: Supplementary file 1 — Supplementary Information [file 42004_2026_2003_MOESM1_ESM.pdf]

Supplementary Information: A detailed comparison of ΔSCF methods with the constraint-based orbital-optimized excited state method

Yannick Lemke,[1,a)] Jörg Kussmann,[1,a)] and Christian Ochsenfeld[1,2]

[1)] *Chair of Theoretical Chemistry, Department of Chemistry, Ludwig-Maximilians-Universität München, Butenandtstr. 5–13, D-81377 Munich, Germany*

[2)] *Max-Planck-Institute for Solid State Research, Heisenbergstr. 1, D-70569 Stuttgart, Germany*

(*Electronic mail: christian.ochsenfeld@uni-muenchen.de)

(*Electronic mail: joerg.kussmann@uni-muenchen.de)

[a)] These authors contributed equally

ADDITIONAL ILLUSTRATIONS AND RESULTS FOR BENZENE

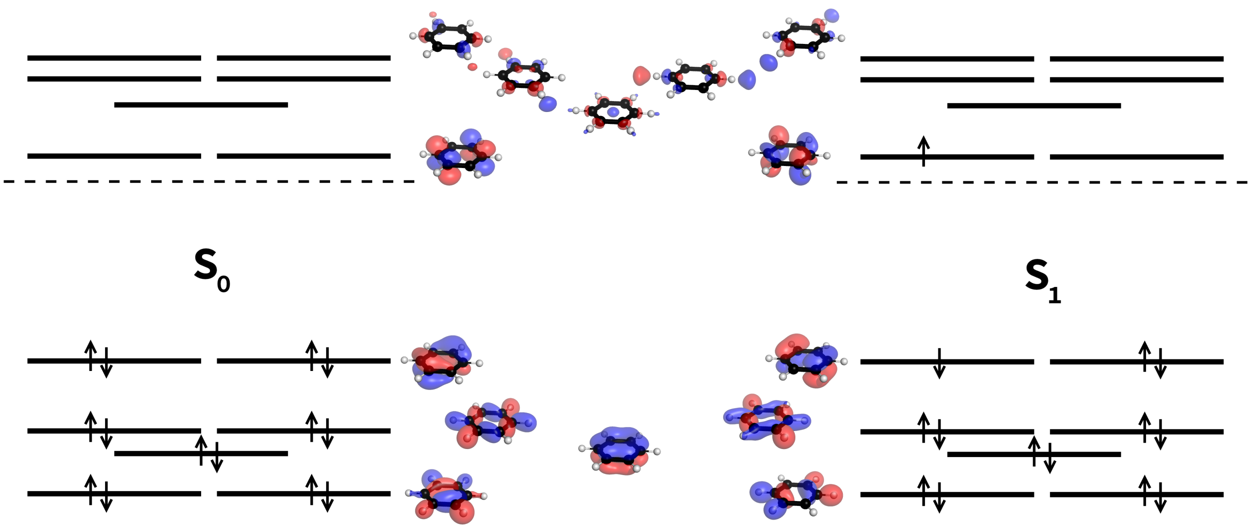

Supplementary Fig. 1. Manual violation of the Aufbau principle to enforce a HOMO $\rightarrow$ LUMO excitation at the example of benzene (PBE0/def2-TZVP). Only the MOs from HOMO $-$ 6 to LUMO $+$ 6 are shown.

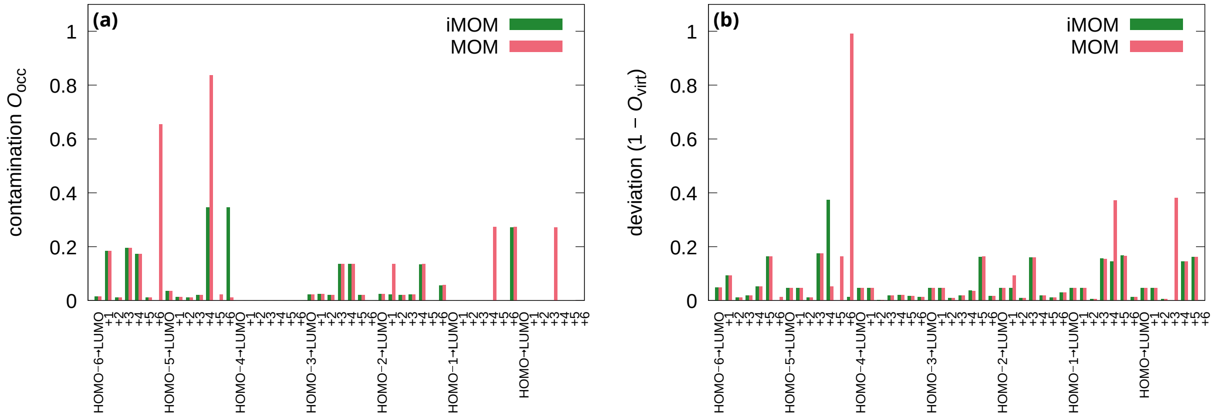

Supplementary Fig. 2. (a) Contamination ($2\,\mathrm{Tr}\,[\mathbf{P}^{\mathrm{exc}}\mathbf{S}\boldsymbol{\Delta}\mathbf{P}^{\mathrm{occ}}\mathbf{S}]$) and (b) deviation ($1 - 2\,\mathrm{Tr}\left[\mathbf{P}^{\mathrm{exc}}\mathbf{S}\boldsymbol{\Delta}\mathbf{P}^{\mathrm{virt}}\mathbf{S}\right]$) of the excited state density of benzene (PBE0/def2-TZVP). By definition, ΔCOOX contamination/deviation is zero.

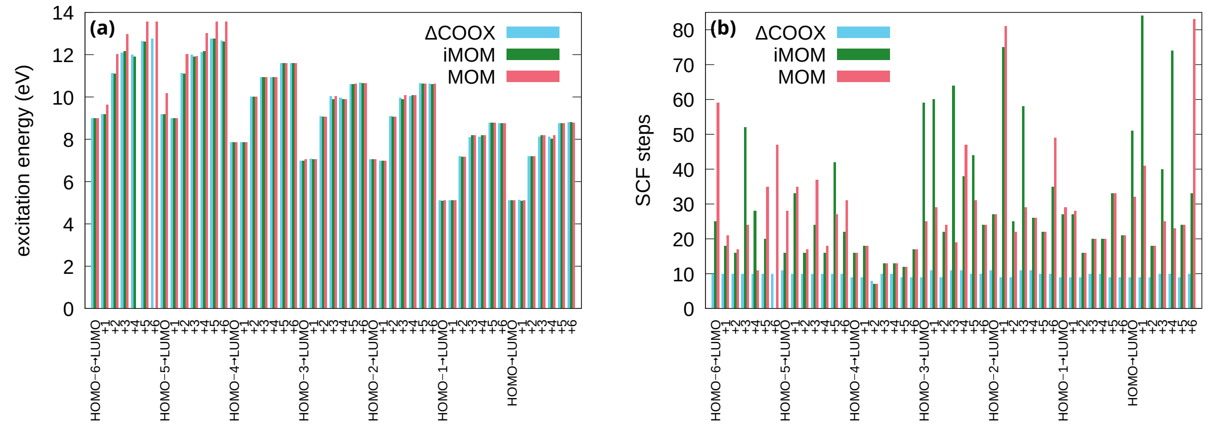

Supplementary Fig. 3. (a) Energies and (b) number of SCF iterations for all excitations HOMO $-$ $x \to$ LUMO $+\,y$ $(x, y = 0, \dots, 6)$ of benzene (PBE/def2-TZVP) using ΔCOOX, iMOM, and MOM. All ΔCOOX and MOM calculations converged, whereas one iMOM calculation failed to converge.

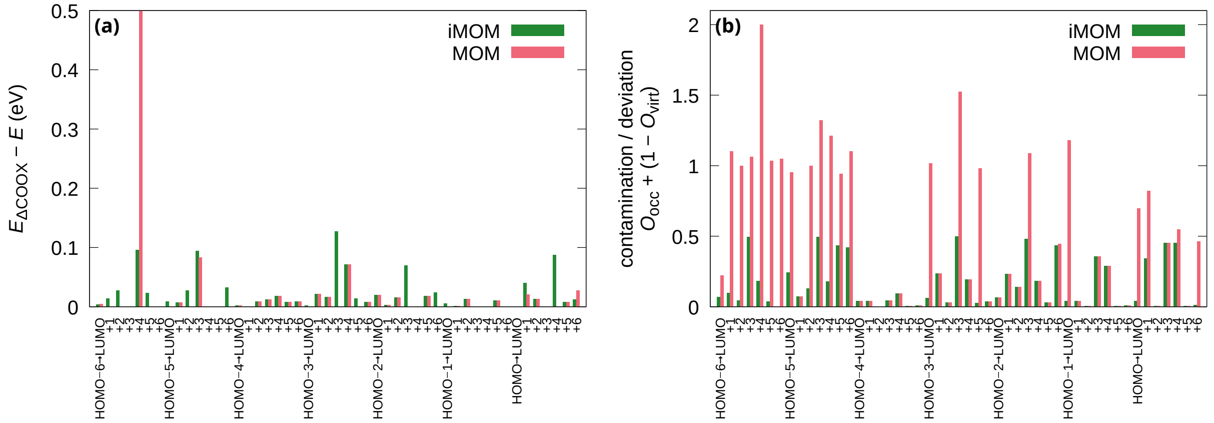

Supplementary Fig. 4. (a) Difference energy relative to ΔCOOX result for all excitations HOMO $-$ $x \to$ LUMO $+\,y$ $(x, y = 0, \dots, 6)$ of benzene (PBE/def2-TZVP). (b) Contamination/deviation of the excited state densities (see text for details) as the sum of the projection onto the single electron density formed from the depleted occupied orbital ϕ_i^α and the deviation from one of the projection onto the corresponding density formed from the filled virtual orbital ϕ_a^α. By definition, contamination/deviation for ΔCOOX is zero. Unconverged MOM/iMOM results are neglected.

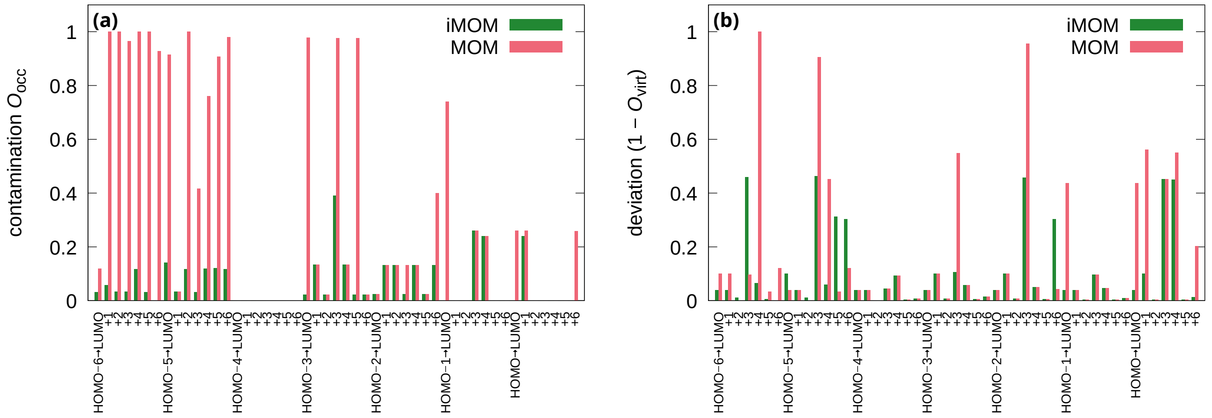

Supplementary Fig. 5. (a) Contamination ($2\,\mathrm{Tr}\,[\mathbf{P}^{\mathrm{exc}}\mathbf{S}\boldsymbol{\Delta}\mathbf{P}^{\mathrm{occ}}\mathbf{S}]$) and (b) deviation ($1 - 2\,\mathrm{Tr}\left[\mathbf{P}^{\mathrm{exc}}\mathbf{S}\boldsymbol{\Delta}\mathbf{P}^{\mathrm{virt}}\mathbf{S}\right]$) of the excited state density of benzene (PBE/def2-TZVP). By definition, ΔCOOX contamination/deviation is zero.

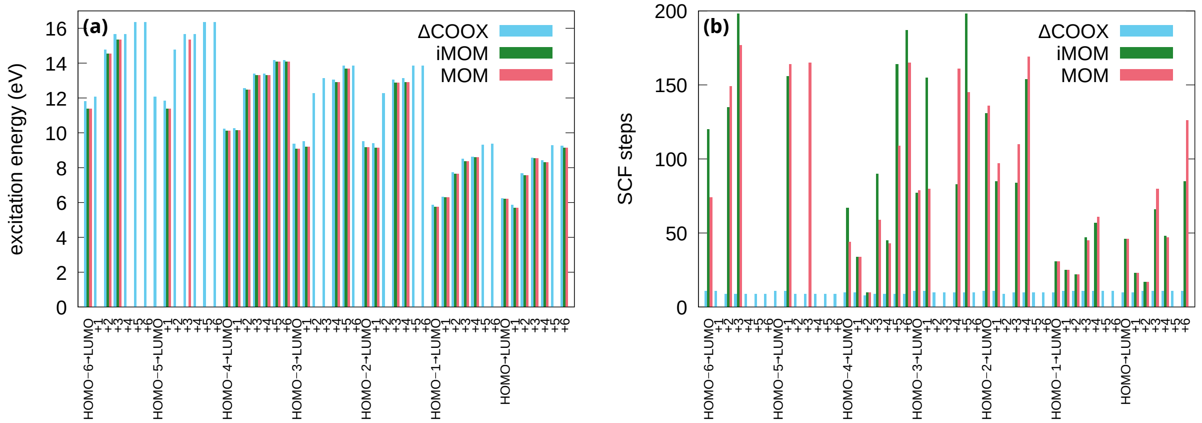

Supplementary Fig. 6. (a) Energies and (b) number of SCF iterations for all excitations HOMO − $x \to$ LUMO + y ($x, y = 0, \ldots, 6$) of benzene (HF/def2-TZVP) using ΔCOOX, iMOM, and MOM. All ΔCOOX calculations converged, whereas 18 MOM and 19 iMOM calculations failed to converge.

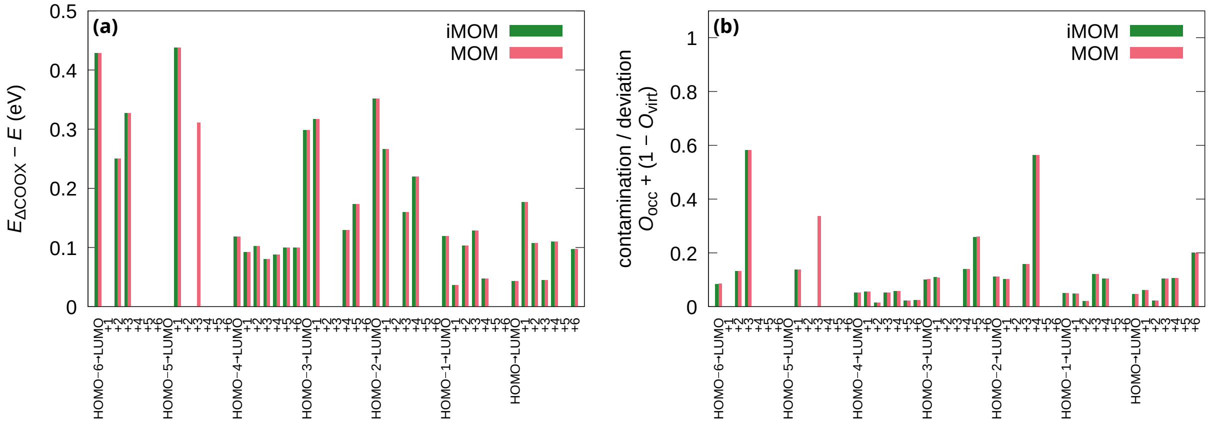

Supplementary Fig. 7. (a) Difference energy relative to ΔCOOX result for all excitations HOMO $- x \rightarrow$ LUMO $+ y$ $(x, y = 0, \ldots, 6)$ of benzene (HF/def2-TZVP). (b) Contamination/deviation of the excited state densities (see text for details) as the sum of the projection onto the single electron density formed from the depleted occupied orbital ϕ_i^α and the deviation from one of the projection onto the corresponding density formed from the filled virtual orbital ϕ_a^α. By definition, contamination/deviation for ΔCOOX is zero. Unconverged MOM/iMOM results are neglected.

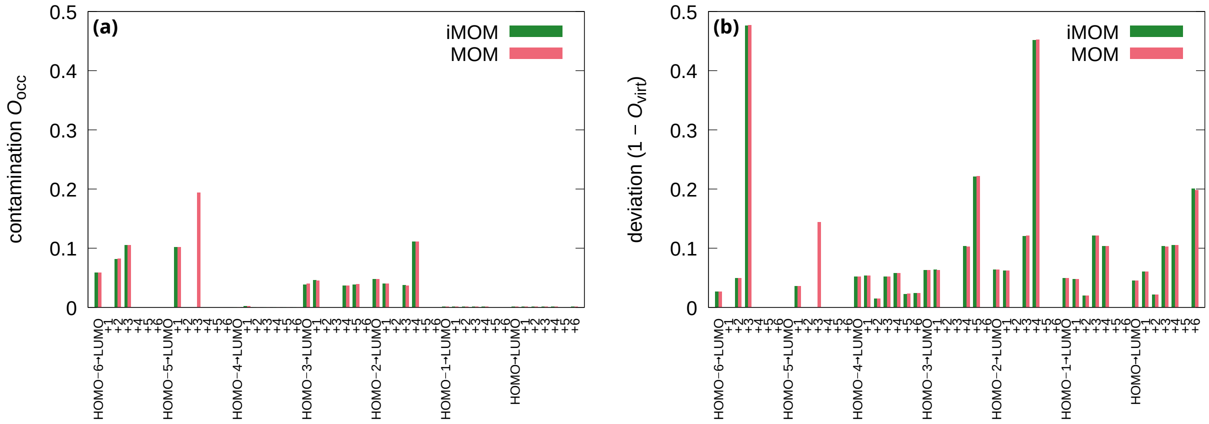

Supplementary Fig. 8. (a) Contamination $(2\,\mathrm{Tr}\left[\mathbf{P}^{\text{exc}}\mathbf{S}\boldsymbol{\Delta}\mathbf{P}^{\text{occ}}\mathbf{S}\right])$ and (b) deviation $(1 - 2\,\mathrm{Tr}\left[\mathbf{P}^{\text{exc}}\mathbf{S}\boldsymbol{\Delta}\mathbf{P}^{\text{virt}}\mathbf{S}\right])$ of the excited state density of benzene (HF/def2-TZVP). By definition, ΔCOOX contamination/deviation is zero.

CHARGE-TRANSFER EXCITATIONS

Supplementary Note 1: Long-range CT in C_2H_4–C_2F_4 for different functionals

In Supplementary Fig. 9, we show the ΔCOOX results when constraining only the α-density. As discussed in the main text, this approach collapses to an energetically lower ^{1}TT state that closely resembles the corresponding ^{1}TT state obtained with cDFT with spatial spin constraints, i.e., the charge difference between the two fragments goes to zero whereas the spin difference approaches a value of 2. Supplementary Fig. 10 shows the equivalent results to Fig. 3 in the main text for the C_2H_4–C_2F_4 CT excitation for different functionals. For the ΔCOOX calculations with the PBE and PBE0 functionals, Fermi smearing at an electronic temperature of 1000 K was used to facilitate convergence, leading to a charge backflow as discussed in Ref. 1 and the main text of this work.

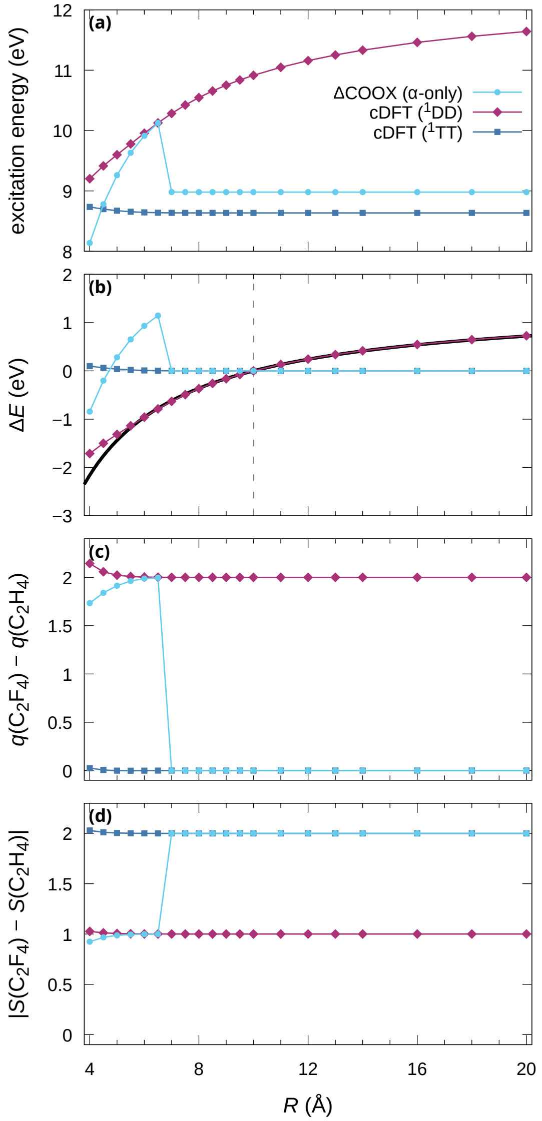

Supplementary Fig. 9. (a) Vertical charge-transfer excitation energies, (b) relative energies aligned at $R = 10\,\text{Å}$ with asymptotic $-1/R$ curve in black, (c) Mulliken charge differences, and (d) total spin differences for C_2H_4–C_2F_4 at different intermolecular separations computed using LRC-ωPBE/def2-TZVP. For ΔCOOX, only the α-density was constrained.

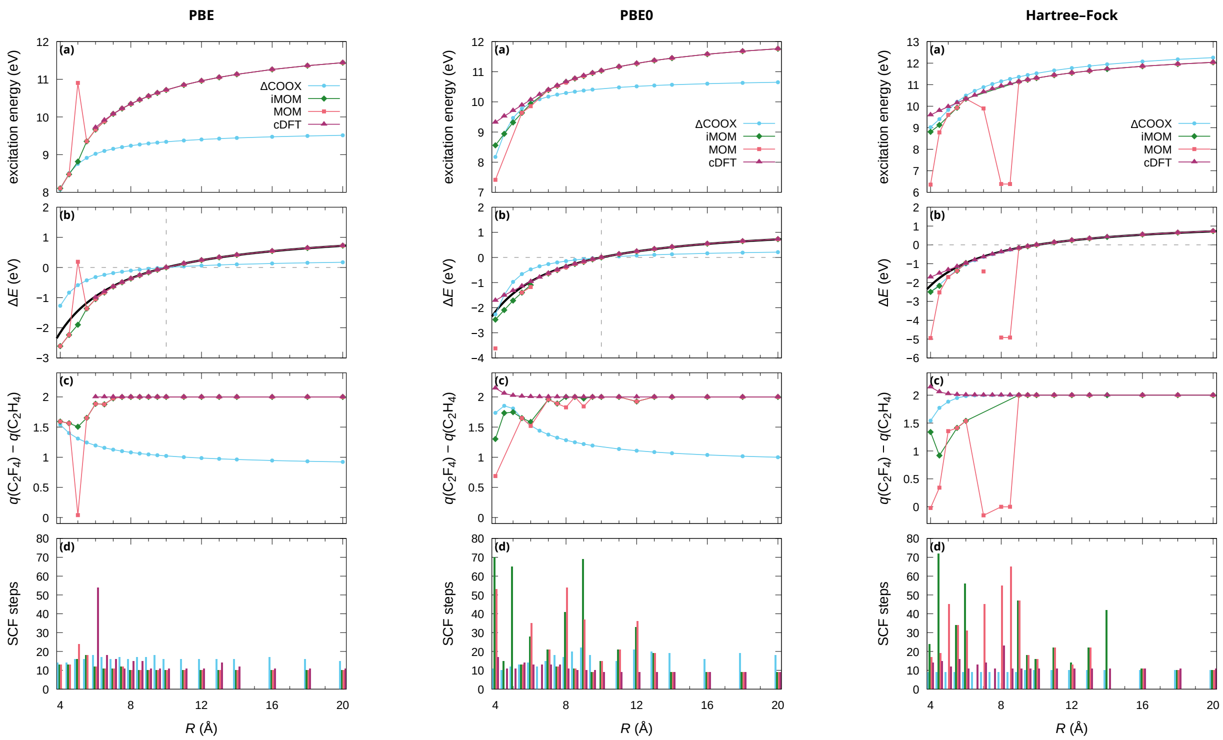

Supplementary Fig. 10. (a) Vertical charge-transfer excitation energies, (b) relative energies aligned at $R = 10$ Å with asymptotic $-1/R$ curve in black, (c) Mulliken charge differences, and (d) number of SCF steps for C_2H_4–C_2F_4 at different intermolecular separations computed using PBE/def2-TZVP (left), PBE0/def2-TZVP (middle), and HF/def2-TZVP (right).

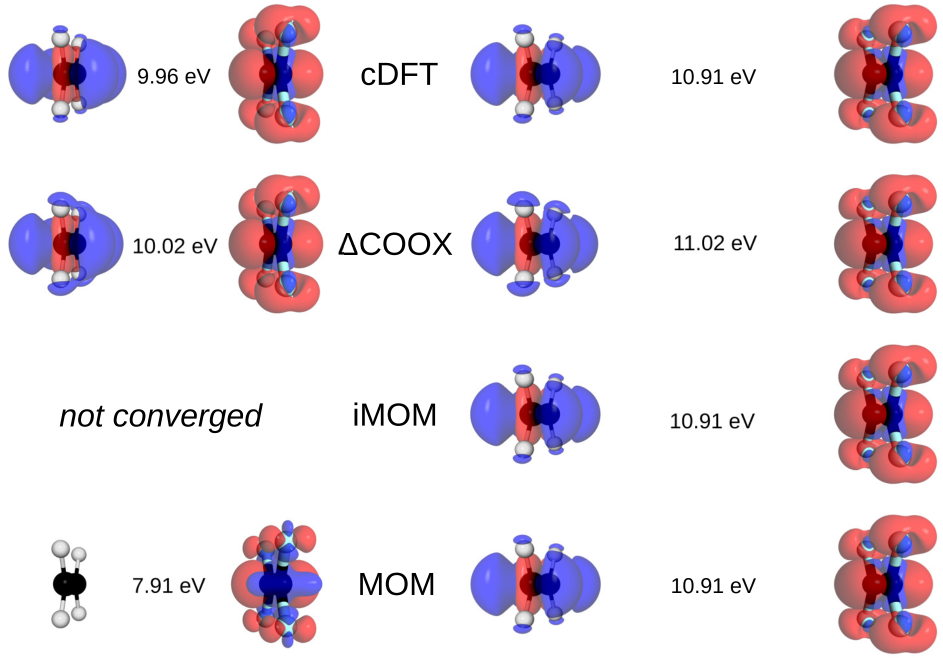

Supplementary Fig. 11. Excited-state difference densities and excitation energies for the intermolecular CT state in C_2H_4–C_2F_4 at $R = 6\,\text{Å}$ (left) and $R = 10\,\text{Å}$ (right) computed with LRC-ωPBE/def2-TZVP. Difference densities are drawn at an isovalue of $0.01\,\text{a.u.}$

Supplementary Note 2: Constraint separation for intramolecular CT

As alluded to in the main text, a separation of constraints (as done in intermolecular long-range CT) may often not be necessary for intramolecular CT if there is no competing linear combination of local excitations, or if said local excitations are sufficiently high in energy compared to the CT state. An example of the first case are small systems such as those present in the QUESTDB database, whereas an example of the second case are the medium-sized TADF emitters in the STGABS27 database,[2] which feature distinct donor and acceptor moieties.

In Supplementary Figs. 12 and 13, we show the two highest occupied and lowest unoccupied orbitals localized on the donor and acceptor moieties of the zincbacteriochlorin–bacteriochlorin (ZnBC–BC) system from Ref. 3 and the 9-[1,4]benzoxaborino[2,3,4-*kl*]phenoxaborine-7-yl-1,3,6,8-tetramethyl-9*H*-carbazole (TMCz-BO) system from Ref. 2.

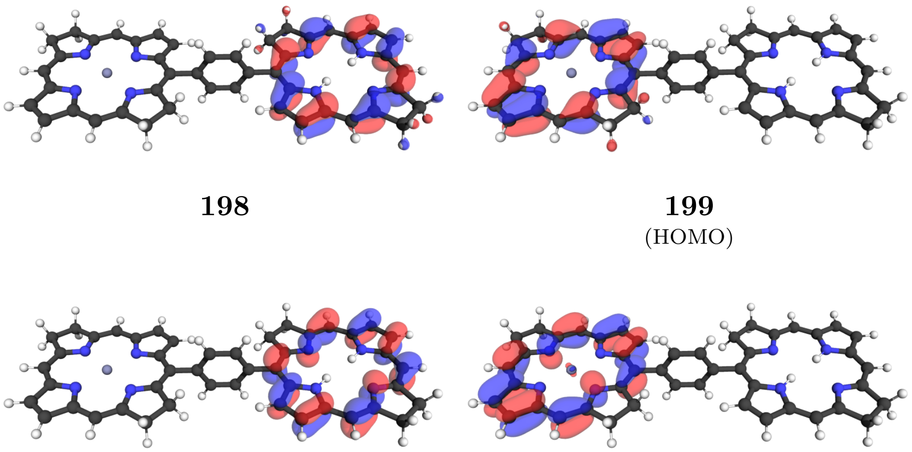

Supplementary Fig. 12. Relevant molecular orbitals for ΔCOOX for the ZnBC–BC system at the LRC-ωPBE/def2-TZVP level, drawn at an isovalue of 0.035 a.u.

The corresponding Supplementary Table 1 lists the obtained ΔCOOX excitation energies for different selections of constraints. In Supplementary Table 1, a constraint specifier (i, a) denotes the constraint potential $\mathbf{S}(\mathbf{C}_a\mathbf{C}_a^\mathsf{T} - \mathbf{C}_i\mathbf{C}_i^\mathsf{T})\mathbf{S}/2$, where $\mathbf{C}_i$ and $\mathbf{C}_a$ are the respective MO column vectors of orbitals i and a. A minus sign, e.g., $-(j, b)$, indicates that the difference density is negated, corresponding to a constraint potential $\mathbf{S}(\mathbf{C}_j\mathbf{C}_j^\mathsf{T} - \mathbf{C}_b\mathbf{C}_b^\mathsf{T})\mathbf{S}/2$ to enforce that orbital j remains occupied and orbital b remains unoccupied. We investigate

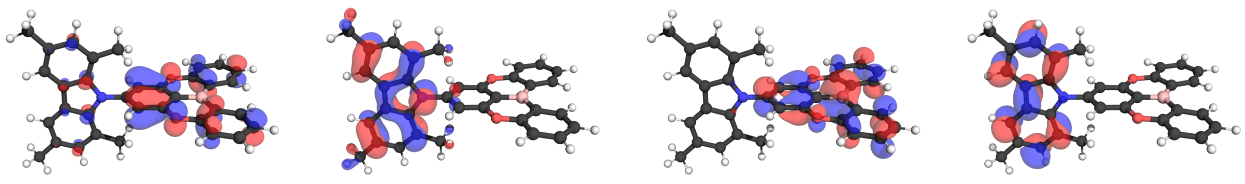

127 **129** (HOMO) **130** (LUMO) **133**

Supplementary Fig. 13. Relevant molecular orbitals for ΔCOOX for the TMCz-BO system at the OT-LRC-ωPBE ($\omega = 0.175\, a_0^{-1}$)/def2-TZVP/IEF-PCM (toluene, $\varepsilon = 2.3741$) level, drawn at an isovalue of 0.035 a.u.

three cases, i.e., targeting the CT state by constraining only the α-spin sector ("simple"), targeting the CT state through a split constraint ("split", cf. intermolecular CT), and explicitly targeting the ^{1}TT-like superposition of local excitations ("double").

Supplementary Table 1. Constraint compositions, excitation energies, and virtual space projections for different ΔCOOX calculations on the TMCz-BO and ZnBC–BC systems.

	TMCz-BO[a]			ZnBC–BC[b]		
constraint	single	split	double	single	split	double
$\mathbf{W}_c^\alpha$	$(129, 130)$	$(129, 130)$	$(129, 130)$	$(199, 200)$	$(199, 200)$	$(199, 200)$
$\mathbf{W}_c^\beta$	—	$-(127, 133)$	$(127, 133)$	—	$-(198, 201)$	$(198, 201)$
ΔE (eV)	2.93	2.95	6.59	1.33	2.62	1.41
# electrons[c]	1.06	1.06	2.04	2.11	1.15	2.11

[a] OT-LRC-ωPBE ($\omega = 0.175\, a_0^{-1}$), def2-TZVP basis set, IEF-PCM (toluene, $\varepsilon = 2.3741$), geometry from Ref. 2. [b] LRC-ωPBE ($\omega = 0.3\, a_0^{-1}$), def2-TZVP basis set, geometry from Ref. 3. [c] Trace of the projection of the excited-state density onto the virtual space of the ground-state.

Evidently, for TMCz-BO, the competing double-excitation is substantially higher in energy than the desired CT state, which is already correctly obtained by constraining only the α-spin sector – a split constraint is of course feasible and does not appear detrimental, yielding virtually identical results. In contrast, the undesired ^{1}TT-like state is obtained for the ZnBC–BC system when using the unmodified ("simple") constraint potential due to low-lying local triplet excitations in the donor and acceptor moieties. Further analysis of the projection onto the ground-state virtual space shows the spurious excitation from orbital

198 (HOMO − 1) to 201 (LUMO + 1) in the β-spin channel, and the resulting wavefunction clearly resembles a triplet state with $\langle \hat{S}^2 \rangle = 2.11$. Similarly to intermolecular CT, adding a constraint potential which inhibits charge backflow in the β-spin sector (i.e., the "split" constraint) remedies this deficiency. Fortunately, as stated in the main text, a given state can be verified by straightforward metrics: When the projection onto the virtual space of the ground-state yields approximately one electron and $\langle \hat{S}^2 \rangle \approx 1$, the obtained state is likely the desired CT state. If instead two electrons are excited and $\langle \hat{S}^2 \rangle \approx 2$, the undesired ^{1}TT-like state was obtained and a split constraint should be considered.

Supplementary Note 3: QUESTDB results for intramolecular CT

In Supplementary Tables 2 and 3, the spin-contaminated and spin-purified excitation energies corresponding to Fig. 6 of the main text are listed. Note that unconverged iMOM results are excluded from the statistics. Values present in Supplementary Table 2 may be missing in Supplementary Table 3 if the corresponding triplet calculation did not converge. The statistical quantities listed are

- Mean (signed) error (**ME**): $\frac{1}{n}\sum_{i=1}^{n} x_i - r_i$
- Mean absolute error (**MAE**): $\frac{1}{n}\sum_{i=1}^{n} |x_i - r_i|$
- (Bessel-corrected) standard deviation of the error (**SDE**): $\sqrt{\frac{1}{n-1}\sum_{i=1}^{n}((x_i - r_i) - \mathrm{ME})^2}$
- Root-mean-squared error (**RMSE**): $\sqrt{\frac{1}{n}\sum_{i=1}^{n}(x_i - r_i)^2}$
- Maximal positive deviation (**max(+)**): $\max\limits_{x_i - r_i > 0} x_i - r_i$
- Maximal negative deviation (**max(−)**): $\min\limits_{x_i - r_i < 0} x_i - r_i$
- Maximal absolute deviation (**AMAX**): $\max |x_i - r_i|$
- Minimal absolute deviation (**AMIN**): $\min |x_i - r_i|$

where n is the number of entries in the benchmark, x_i are the computed excitation energies, and r_i are the theoretical best estimates (TBE).

Supplementary Table 2: Spin-contaminated excitation energies and statistical quantities for the intramolecular CT benchmark of the QUEST database,[4] computed using iMOM and ΔCOOX with the def2-TZVP basis set and different functionals. All values in eV.

molecule	state[a]	TBE	iMOM				ΔCOOX			
			PBE	PBE0	LRC-ωPBE	ωB97X	PBE	PBE0	LRC-ωPBE	ωB97X
ABN	2 A_1	5.09	3.72	4.12	4.46	4.62	3.76	4.14	4.54	4.69
aniline	2 A_1	5.48	4.39	4.74	4.98	5.14	4.43	4.77	5.04	5.21
azulene	2 A_1	3.84	3.01	3.27	3.54	3.64	3.01	3.27	3.54	3.65
	2 B_2	4.49	3.98	4.17	4.32	4.40	3.98	4.17	4.33	4.42
benzonitrile	1 A_2	7.05	5.93	6.32	6.53	6.55	6.48	6.63	6.83	6.96
BTD	1 B_2	4.28	3.08	3.34	3.56	3.63	3.15	3.35	3.57	3.66
DMABN	2 A_1	4.86	3.56	3.94	4.30	4.46	3.63	3.97	4.37	4.52
DMAn	1 B_2	4.40	3.86	4.11	4.29	4.41	3.98	4.15	4.36	4.49
	2 A_1	5.40	4.21	4.57	4.84	5.01	4.27	4.60	4.90	5.07
dipeptide	7 A''	8.15	7.35	7.60	6.62	6.97	7.29	7.26	7.51	7.62
β-dipeptide	7 A'	8.51	7.54	7.89	—	7.70	7.09	7.26	7.73	7.92
	10 A''	8.90	7.80	8.21	—	7.37	7.79	7.72	8.08	8.17
HCl	1 Π	7.88	7.54	7.60	7.65	7.90	7.57	7.68	7.81	8.07
nitroaniline	2 A_1	4.39	3.32	3.59	3.95	4.04	3.67	3.75	4.07	4.10
nitrobenzene	2 A_1	5.39	5.27	4.44	4.72	4.83	5.28	4.56	4.86	4.89
NDMAn	2 A_1	4.13	3.12	3.38	3.76	3.84	3.49	3.54	3.86	3.89
NPNO	2 A_1	4.10	2.75	2.94	3.30	3.37	2.97	3.05	3.39	3.40
PP	2 B_2	5.32	4.16	4.52	4.73	4.85	4.58	4.77	5.00	5.09
	3 A_1	5.86	5.19	5.23	5.70	5.75	5.49	5.56	5.71	5.77
phthalazine	1 A_2	3.91	3.05	3.45	3.48	3.75	3.23	3.62	3.70	3.99
	1 B_1	4.31	3.39	3.73	3.73	3.96	3.56	3.91	3.95	4.21
quinoxaline	1 B_2	4.63	3.49	3.79	4.01	4.09	3.60	3.81	4.03	4.13
	3 A_1	5.65	4.57	4.90	5.26	5.36	4.60	4.91	5.32	5.42

(continued on next page)

Supplementary Table 2: (continued)

molecule	state[a]	TBE	iMOM				ΔCOOX			
			PBE	PBE0	LRC-ωPBE	ωB97X	PBE	PBE0	LRC-ωPBE	ωB97X
	2 B_1	6.22	5.16	5.88	6.22	6.59	5.11	5.83	6.21	6.60
twisted DMABN	1 A_2	4.12	3.60	3.76	3.86	3.98	3.88	3.96	4.05	4.19
	1 B_1	4.75	4.27	4.47	4.56	4.69	4.45	4.55	4.63	4.78
twisted PP	2 B_2	5.58	5.31	5.02	5.58	5.43	5.38	5.39	5.60	5.51
	2 A_1	5.65	5.08	5.12	5.37	5.63	5.48	5.51	5.45	5.65
	1 A_2	5.95	5.43	5.70	6.05	5.92	5.85	5.92	6.13	6.07
	1 B_1	6.17	5.45	5.84	5.82	6.15	5.98	6.06	5.96	6.23
ME			−0.86	−0.63	−0.42	−0.35	−0.71	−0.56	−0.33	−0.20
MAE			0.86	0.63	0.43	0.38	0.71	0.56	0.34	0.27
SDE			0.34	0.25	0.32	0.38	0.40	0.34	0.26	0.28
RMSE			0.93	0.67	0.53	0.51	0.82	0.65	0.42	0.34
max(+)			—	—	0.10	0.37	—	—	0.18	0.38
max(−)			−1.37	−1.16	−1.53	−1.53	−1.42	−1.25	−0.82	−0.73
AMAX			1.37	1.16	1.53	1.53	1.42	1.25	0.82	0.73
AMIN			0.12	0.25	0.00	0.01	0.10	0.03	0.01	0.00
**statistics excluding dipeptide and β-dipeptide**										
ME			−0.85	−0.63	−0.38	−0.26	−0.67	−0.50	−0.28	−0.16
MAE			0.85	0.63	0.38	0.29	0.67	0.50	0.30	0.23
SDE			0.35	0.26	0.24	0.25	0.39	0.30	0.23	0.26
RMSE			0.92	0.68	0.45	0.35	0.77	0.58	0.36	0.30
max(+)			—	—	0.10	0.37	—	—	0.18	0.38
max(−)			−1.37	−1.16	−0.80	−0.73	−1.33	−1.05	−0.71	−0.70
AMAX			1.37	1.16	0.80	0.73	1.33	1.05	0.71	0.70
AMIN			0.12	0.25	0.00	0.01	0.10	0.03	0.01	0.00

[a]State ordering according to Ref. 4, may not necessarily reflect the iMOM or ΔCOOX state ordering.

Supplementary Table 3: Spin-purified excitation energies and statistical quantities for the intramolecular CT benchmark of the QUEST database,[4] computed using iMOM and ΔCOOX with the def2-TZVP basis set and different functionals. All values in eV.

molecule	state[a]	TBE	iMOM				ΔCOOX			
			PBE	PBE0	LRC-ωPBE	ωB97X	PBE	PBE0	LRC-ωPBE	ωB97X
ABN	2 A_1	5.09	4.01	4.67	5.28	5.49	3.98	4.61	5.25	5.46
aniline	2 A_1	5.48	4.78	5.44	5.87	6.10	4.73	5.36	5.82	6.04
azulene	2 A_1	3.84	3.33	3.87	4.45	4.58	3.30	3.84	4.42	4.55
	2 B_2	4.49	4.09	4.39	4.66	4.77	4.09	4.38	4.66	4.77
benzonitrile	1 A_2	7.05	5.95	6.31	6.50	6.53	6.47	6.57	6.75	6.87
BTD	1 B_2	4.28	3.41	3.88	4.15	4.27	3.41	3.80	4.13	4.26
DMABN	2 A_1	4.86	3.79	4.40	5.03	5.24	3.76	4.32	4.96	5.17
DMAn	1 B_2	4.40	4.00	4.35	4.63	4.77	4.07	4.32	4.59	4.72
	2 A_1	5.40	4.52	5.14	5.64	5.86	4.47	5.04	5.54	5.76
dipeptide	7 A''	8.90	7.53	7.49	6.59	6.91	7.07	7.16	7.44	7.93
β-dipeptide	7 A'	8.15	7.78	—	—	—	7.71	7.23	7.73	8.08
	10 A''	8.51	7.31	—	—	—	7.22	7.62	8.00	7.53
HCl	1 Π	7.88	7.76	7.82	7.86	8.12	7.75	7.83	7.94	8.20
nitroaniline	2 A_1	4.39	3.54	3.96	4.55	4.64	3.58	3.86	4.41	4.48
nitrobenzene	2 A_1	5.39	5.43	5.02	5.07	5.61	5.42	4.85	5.34	5.42
NDMAn	2 A_1	4.13	3.29	3.67	4.29	4.38	3.35	3.60	4.14	4.20
NPNO	2 A_1	4.10	3.06	3.61	4.28	4.46	2.95	3.41	4.03	4.14
PP	2 B_2	5.32	4.52	5.17	5.90	—	4.72	4.97	5.17	5.26
	3 A_1	5.86	4.63	5.02	—	—	5.40	5.41	5.56	5.61
phthalazine	1 A_2	3.91	3.07	3.40	—	3.65	3.22	3.54	3.60	3.86
	1 B_1	4.31	3.53	3.86	3.83	4.06	3.62	3.92	3.94	4.19
quinoxaline	1 B_2	4.63	3.82	4.29	4.58	4.66	3.81	4.16	4.49	4.61
	3 A_1	5.65	4.66	5.09	5.64	5.75	4.67	5.08	5.66	5.77

(continued on next page)

Supplementary Table 3: (continued)

molecule	state[a]	TBE	iMOM				ΔCOOX			
			PBE	PBE0	LRC-ωPBE	ωB97X	PBE	PBE0	LRC-ωPBE	ωB97X
	$2\,B_1$	6.22	5.16	5.84	6.17	6.53	5.14	5.83	6.20	6.58
twisted DMABN	$1\,A_2$	4.12	3.55	3.65	3.73	3.83	3.81	3.84	3.93	4.05
	$1\,B_1$	4.75	4.23	4.37	4.46	4.57	4.39	4.44	4.52	4.66
twisted PP	$2\,B_2$	5.58	5.27	—	—	—	5.32	5.27	5.48	5.36
	$2\,A_1$	5.65	4.50	—	—	—	5.40	5.38	5.31	5.50
	$1\,A_2$	5.95	5.39	—	—	—	5.76	5.73	5.95	5.84
	$1\,B_1$	6.17	5.55	—	—	—	5.90	5.88	5.76	6.02
ME			−0.77	−0.38	−0.04	0.10	−0.67	−0.44	−0.12	0.01
MAE			0.77	0.38	0.31	0.35	0.67	0.44	0.24	0.25
SDE			0.31	0.25	0.39	0.37	0.37	0.31	0.32	0.34
RMSE			0.83	0.44	0.45	0.44	0.76	0.53	0.33	0.33
max(+)			0.04	0.03	0.61	0.74	0.03	—	0.58	0.71
max(−)			−1.23	−0.84	−1.56	−1.24	−1.44	−1.28	−0.90	−0.82
AMAX			1.23	0.84	1.56	1.24	1.44	1.28	0.90	0.82
AMIN			0.04	0.03	0.01	0.01	0.03	0.00	0.00	0.02
**statistics excluding dipeptide and β-dipeptide**										
ME			−0.74	−0.36	0.04	0.17	−0.61	−0.36	−0.05	0.09
MAE			0.75	0.37	0.26	0.31	0.61	0.36	0.18	0.20
SDE			0.32	0.24	0.28	0.29	0.34	0.18	0.23	0.25
RMSE			0.81	0.42	0.31	0.36	0.69	0.40	0.23	0.26
max(+)			0.04	0.03	0.61	0.74	0.03	—	0.58	0.71
max(−)			−1.23	−0.84	−0.55	−0.52	−1.15	−0.69	−0.41	−0.25
AMAX			1.23	0.84	0.61	0.74	1.15	0.69	0.58	0.71
AMIN			0.04	0.03	0.01	0.01	0.03	0.00	0.00	0.02

[a]State ordering according to Ref. 4, may not necessarily reflect the iMOM or ΔCOOX state ordering.

CORE-EXCITATION ENERGIES

Supplementary Table 4. Core-excitation energies in for a selection of small molecules.

molecule		exp.	ΔCOOX		COOX/CVS-TDA			iMOM		
			ΔE	SCF steps	ΔE	SCF steps	$O_{\mathrm{occ}} + (1 - O_{\mathrm{virt}})$ (T|S)	ΔE	SCF steps	$O_{\mathrm{occ}} + (1 - O_{\mathrm{virt}})$ (T|S)
K-edge excitations of second-period elements										
**C**H_4		288.0[5]	287.6	8	287.5	9	0.197 |0.079	287.3	47	0.422 |0.374
**N**H_3		400.8[5]	400.5	8	400.4	9	0.126 |0.046	400.1	9	0.196 |0.165
H_2**O**		534.0[5]	534.0	9	533.8	9	0.149 |0.063	533.4	9	0.224 |0.196
H**F**		687.4[6]	687.8	8	687.3	8	0.186 |0.091	686.9	9	0.256 |0.233
L-edge excitations of third-period elements										
**Si**H_4	L_3	102.6[7]	103.4	8	103.1	8	0.105 |0.067	102.8	19	1.226 |0.217
	L_2	103.2[7]	104.0		103.7			103.4		
**P**H_3	L_3	131.9[8]	132.5	10	132.0	10	0.111 |0.098	131.9	20	0.195 |0.180
	L_2	132.8[8]	133.3		132.8			132.7		
H_2**S**	L_3	164.4[9]	165.5	9	165.0	9	0.140 |0.119	164.8	9	0.250 |0.246
	L_2	165.6[9]	166.7		166.2			166.0		
H**Cl**	L_3	200.6[10]	202.0	8	202.1	8	0.256 |0.214	201.0	18	0.305 |0.292
	L_2	202.4[10]	203.6		202.8			202.6		
L- and M-edge excitations of heavy elements										
**Cr**O_2Cl_2	L_3	579.9[11]	578.2	26	578.5	25	0.051 |0.125	578.4	27	0.075 |0.031
	L_2	588.5[11]	586.8		587.1			587.0		
**Mo**S_4^{2-}	M_5	228.7[12]	232.7	16	232.1	16	0.509 |0.525	n.c.		
	M_4	231.7[12]	235.7		235.1			n.c.		
	L_3	2521.6[12]	2537.3	17	2536.4	17	0.409 |0.405	n.c.		
	L_2	2620.6[12]	2636.2		2635.3			n.c.		
**W**Cl_6	L_3	10212.2[13]	10283.9	17	10283.8	16	0.816 |0.902	n.c.		
	L_2	11547.1[13]	11618.8		11618.6			n.c.		

All energies in eV, computed with the PBE0 functional. For COOX/CVS-TDA and iMOM, the constraint contamination/deviation relative to ΔCOOX is also shown, for L- and M-edge excitations, these represent the maximal value among the excitation from the three p- or five d-orbitals, respectively. Basis sets used: aug-pcX-2 for CH_4 through HCl, aug-cc-pwCVTZ-DK for CrO_2Cl_2 and MoS_4^{2-}, ma-ZORA-def2-TZVPP/SARC-ZORA-TZVPP for WCl_6.

SUPPLEMENTARY REFERENCES

[1] J. Kussmann, Y. Lemke, A. Weinbrenner, and C. Ochsenfeld, "A constraint-based orbital-optimized excited state method (COOX)," J. Chem. Theory Comput. **20**, 8461–8473 (2024).

[2] L. Kunze, A. Hansen, S. Grimme, and J.-M. Mewes, "PCM-ROKS for the description of charge-transfer states in solution: Singlet–triplet gaps with chemical accuracy from open-shell Kohn–Sham reaction-field calculations," J. Phys. Chem. Lett. **12**, 8470–8480 (2021).

[3] A. Dreuw and M. Head-Gordon, "Failure of time-dependent density functional theory for long-range charge-transfer excited states: The zincbacteriochlorin–bacteriochlorin and bacteriochlorophyll–spheroidene complexes," J. Am. Chem. Soc. **126**, 4007–4016 (2004).

[4] P.-F. Loos, M. Comin, X. Blase, and D. Jacquemin, "Reference energies for intramolecular charge-transfer excitations," J. Chem. Theory Comput. **17**, 3666–3686 (2021).

[5] J. Schirmer, A. B. Trofimov, K. J. Randall, J. Feldhaus, A. M. Bradshaw, Y. Ma, C. T. Chen, and F. Sette, "K-shell excitation of the water, ammonia, and methane molecules using high-resolution photoabsorption spectroscopy," Phys. Rev. A **47**, 1136–1147 (1993).

[6] A. P. Hitchcock and C. E. Brion, "K-shell excitation of HF and F_2 studied by electron energy-loss spectroscopy," J. Phys. B: At. Mol. Phys. **14**, 4399 (1981).

[7] W. Hayes and F. C. Brown, "Absorption by some molecular gases in the extreme ultraviolet," Phys. Rev. A **6**, 21–30 (1972).

[8] Z. F. Liu, J. N. Cutler, G. M. Bancroft, K. H. Tan, R. G. Cavell, and J. S. Tse, "High resolution gas phase photoabsorption spectra and multiple-scattering Xα study of PX_3 (X = H , CH_3, CF_3) compounds at the P $L_{2,3}$ edge," Chem. Phys. Lett. **172**, 421–429 (1990).

[9] R. Guillemin, W. C. Stolte, L. T. N. Dang, S.-W. Yu, and D. W. Lindle, "Fragmentation dynamics of H_2S following S 2p photoexcitation," J. Chem. Phys. **122**, 094318 (2005).

[10] H. Aksela, S. Aksela, M. Ala-Korpela, O.-P. Sairanen, M. Hotokka, G. M. Bancroft, K. H. Tan, and J. Tulkki, "Decay channels of core-excited HCl," Phys. Rev. A **41**, 6000–6005 (1990).

[11] G. Fronzoni, M. Stener, P. Decleva, M. d. Simone, M. Coreno, P. Franceschi, C. Furlani, and K. C. Prince, "X-ray absorption spectroscopy of $VOCl_3$, CrO_2Cl_2, and MnO_3Cl: An experimental and theoretical study," J. Phys. Chem. A **113**, 2914–2925 (2009).

[12]S. J. George, O. B. Drury, J. Fu, S. Friedrich, C. J. Doonan, G. N. George, J. M. White, C. G. Young, and S. P. Cramer, “Molybdenum X-ray absorption edges from 200 to 20,000 eV: The benefits of soft X-ray spectroscopy for chemical speciation,” J. Inorg. Biochem. **103**, 157–167 (2009).

[13]U. Jayarathne, P. Chandrasekaran, A. F. Greene, J. T. Mague, S. DeBeer, K. M. Lancaster, S. Sproules, and J. P. Donahue, “X-ray absorption spectroscopy systematics at the tungsten L-edge,” Inorg. Chem. **53**, 8230–8241 (2014).
