# Supplementary material for: A detailed comparison of ΔSCF methods with the constraint-based orbital-optimized excited state method
Source: Commun Chem. 2026 Apr 22;9:162. doi: 10.1038/s42004-026-02003-9 (PMC13102931; doi:10.1038/s42004-026-02003-9)
Supplement: Supplementary file 2 — Description of Additional Supplementary Files [file 42004_2026_2003_MOESM2_ESM.pdf]

Description of Additional Supplementary Files:

File: Supplementary Data 1

Description: Geometries of the studied systems in xyz-format.

File: Supplementary Data 2

Description: Energies, number of SCF iterations, contamination/deviation values, and $\langle S^2 \rangle$ values for all computed benzene states.

File: Supplementary Data 3

Description: Energies, number of SCF iterations, contamination/deviation values, and errors relative to TBE for all computed double-excitations.
